# A phosphoinositide switch from PI(4,5)P_2_ to PI4P triggers endocytosis by inducing dynamin-mediated fission in secretory cells

**DOI:** 10.1126/sciadv.ady8065

**Published:** 2025-10-15

**Authors:** Xiaoli Guo, Lisi Wei, Nidhi Kundu, Wonchul Shin, Chung Yu Chan, Sue Han, Ammar Mohseni, Maryam Molakarimi, Min Sun, Xin-Sheng Wu, Yinghui Jin, Jenny E. Hinshaw, Ling-Gang Wu

**Affiliations:** ^1^ National Institute of Neurological Disorders and Stroke, Bethesda, MD, USA.; ^2^ Structural Cell Biology Section, Laboratory of Cell and Molecular Biology, National Institute of Diabetes and Digestive and Kidney Diseases, Bethesda, MD, USA.

## Abstract

Endocytosis generates life-essential vesicles via complex protein-lipid machinery, yet its initiation mechanisms remain elusive. Long thought to require full machinery spatiotemporal coordination to drive the flat-to-round vesicle transformations, we reveal a notably simple initiation mechanism in neuroendocrine chromaffin cells involving only the final step, the pore closure. During calcium-triggered exocytosis, calcium activates the phosphatase synaptojanin, rapidly converting PI(4,5)P_2_ to PI4P. Elevated PI4P drives the fission enzyme dynamin to close the pores of preexisting and exocytosis-generated Ω-profiles, which are sufficient to generate slow, fast, ultrafast, overshoot, and bulk endocytosis, and kiss-and-run (fusion pore closure). These findings resolve the long-standing mystery of endocytosis and fission initiation and reveal PI4P, not the widely assumed PI(4,5)P_2_, as the key lipid for dynamin-mediated fission. This pathway is clinically important, as it is impaired by synaptojanin mutations associated with Parkinson’s disease and seizures, and is likely disrupted in other disorders involving PI4P or PI(4,5)P_2_ dysregulation.

## Teaser

Calcium activates synaptojanin to convert PI(4,5)P_2_ into PI4P, triggering endocytosis by initiating dynamin-mediated fission.

## INTRODUCTION

Endocytosis orchestrates a macromolecular machinery of tens of proteins and lipids that transforms plasma membranes (PMs) into vesicles, enabling essential cellular functions such as vesicle recycling, synaptic transmission, nutrient uptake, intracellular trafficking, and viral infection (*1*–*6*). This process occurs rapidly, often within seconds or milliseconds, especially in neurons and neuroendocrine cells, where fast or ultrafast endocytosis must be initiated after exocytosis to recycle vesicles and sustain synaptic transmission in a process known as exo-endocytosis coupling (*4*, *5*, *7*). Despite over 50 years of study since the discovery of endocytosis at synapses, the mechanism that rapidly initiates the endocytic machinery to meet urgent physiological demands, including vesicle recycling to sustain the exocytosis capacity, remains unclear (*1*, *4*, *8*). It has long been implicitly assumed that a yet unidentified signal coordinates this complex macromolecular machinery in a spatiotemporal pattern, converting the membrane from a flat structure to a dome- or Λ-shaped intermediate and eventually to a Ω- and then O-shaped vesicle. This assumption makes it seem highly challenging for a single signal to activate tens of proteins and lipids in such a precise pattern. Identifying this signal to coordinate the entire endocytic macromolecular machinery has proven equally difficult, a mystery that remains unresolved to this day.

Recent studies in neuroendocrine chromaffin cells have shown that endocytosis following depolarization-induced exocytosis is not primarily driven by the flat-to-round membrane transformation, as previously thought (*4*, *8*). Instead, it is primarily mediated by the final step of endocytosis, the pore closure of the Ω-shape profiles, resulting in the formation of oval/round vesicles (*9*, *10*). The Ω-profiles are either preformed before depolarization (pre-Ω) or generated by depolarization-induced vesicle fusion at the PM (fs-Ω, see also Results for experimental definition) (*9*, *10*). This suggests that triggering the closure of pre-Ω/fs-Ω pores, rather than initiating the entire flat-to-round transformation, could provide a “simpler” yet efficient mechanism for rapidly initiating endocytosis without activating the full machinery responsible for Ω-profile formation. In testing this hypothesis in neuroendocrine secretory cells, we identified a phosphoinositide metabolic pathway that initiates endocytosis: depolarization-induced calcium influx activates the phosphatase synaptojanin, which dephosphorylates phosphatidylinositol 4,5 bisphosphate [PI(4,5)P_2_] into phosphatidylinositol-4-phosphate (PI4P) within milliseconds to seconds. The resulting increase in PI4P promotes the ubiquitous fission enzyme dynamin to mediate pre-Ω/fs-Ω pore closure, which generates diverse modes of endocytosis and kiss-and-run fusion to recycle exocytosed vesicles. This initiation pathway has clinical relevance, as it is disrupted by synaptojanin mutations linked to Parkinson’s disease and seizure, and may also be affected in other disorders involving impaired phosphoinositide metabolism (*11*–*14*).

## RESULTS

**Fig. 1.**
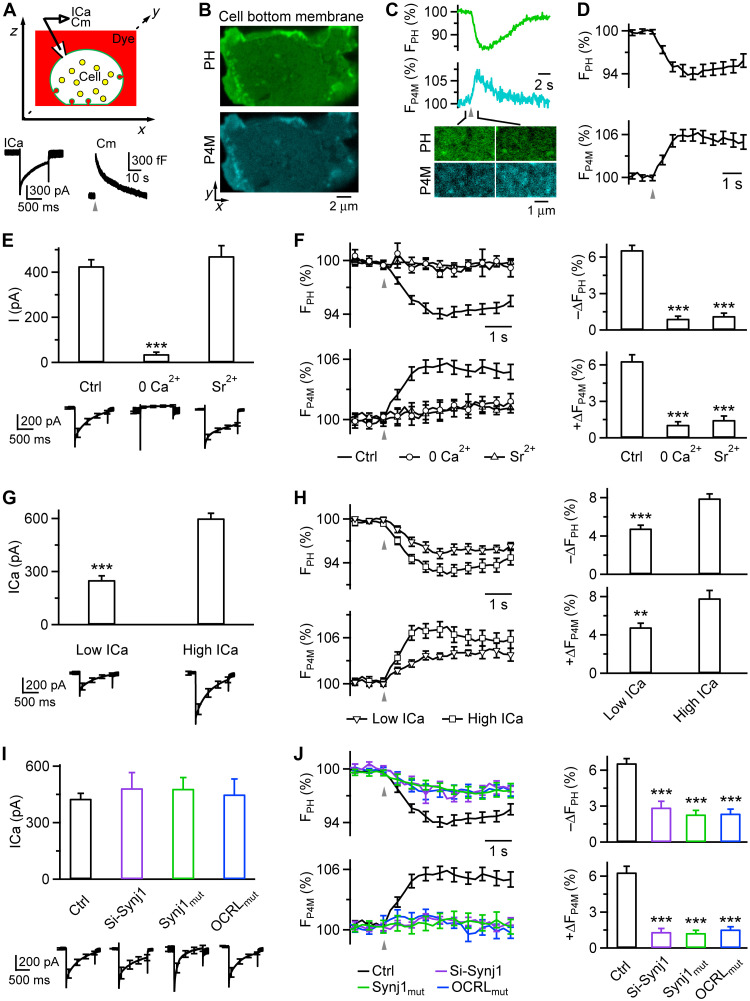
Depolarization-induced calcium influx triggers synaptojanin-mediated PI(4,5)P_2_-to-PI4P transition. (**A**) Upper: Setup drawing—cell membrane, bath, and vesicles are fluorescently labeled; ICa and Cm (capacitance) are recorded (for all figures). Lower: Sampled ICa and Cm induced by depol_1s_ (gray triangle, applies to all panels). (**B**) *XY*-plane confocal images showing PH_mCh_-labeled PI(4,5)P_2_ and P4M-EGFP-labeled PI4P at the cell-bottom PM. (**C**) Depol_1s_-induced PH_mCh_ fluorescence (F_PH_) and P4M-EGFP fluorescence (F_P4M_) and sampled images (cell-bottom, times indicated by sticks). (**D**) Depol_1s_-induced F_PH_ and F_P4M_ from the same cells (mean ± SEM, 26 cells, measured from the entire cell bottom). (**E**) Depol_1s_-induced calcium-channel current peak amplitude (I, mean + SEM) and trace (lower, mean ± SEM) in 5 mM extracellular Ca^2+^ (Ctrl: 52 cells), 0 Ca^2+^ (12 cells), or Sr^2+^ (5 mM, 12 cells). ***P < 0.001, ANOVA; amplitude measured at the maximum. (**F**) Depol_1s_-induced F_PH_ (42, 6, and 6 cells) and F_P4M_ (36, 6, and 6 cells) in Ctrl, 0 Ca^2+^, or Sr^2+^. Left: Traces (mean ± SEM). Right: F_PH_ decrease (−ΔF_PH_) and F_P4M_ increase amplitude (ΔF_P4M_); data (mean + SEM) normalized to baseline; ***P < 0.001, ANOVA (comparing Ctrl). (**G**) Depol_1s_-induced ICa peak amplitude (mean + SEM) and trace (mean ± SEM) in low ICa (< 400 pA, 26 cells) and high ICa group (>400 pA, 26 cells). ***P < 0.001, *t* test. (**H**) Depol_1s_-induced F_PH_ (18 and 18 cells) and F_P4M_ (18 and 24 cells) in low ICa and high ICa group. Left: Traces (mean ± SEM). Right: −ΔF_PH_ and ΔF_P4M_ (mean + SEM). ***P < 0.001, *t* test. (**I**) Depol_1s_-induced ICa amplitude (mean + SEM) and trace (lower, mean ± SEM) in Ctrl (52 cells), synaptojanin 1 Si-RNA–transfected cells (Si-Synj1, 16 cells), synaptojanin 1 R839C-transfected cells (Synj1_mut_, 15 cells), and blue-light-exposed CIBN-CAAX/CRY2-OCRL_mut_–transfected cells (OCRL_mut_, 20 cells). (**J**) Depol_1s_-induced F_PH_ (42, 6, 7, and 11 cells) and F_P4M_ (36, 10, 8, and 9 cells) in Ctrl, Si-Synj1, Synj1_mut_, or OCRL_mut_. Left: Traces (mean ± SEM). Right: −ΔF_PH_ and ΔF_P4M_ (mean + SEM). ***P < 0.001, ANOVA (comparing Ctrl).

**Fig. 2.**
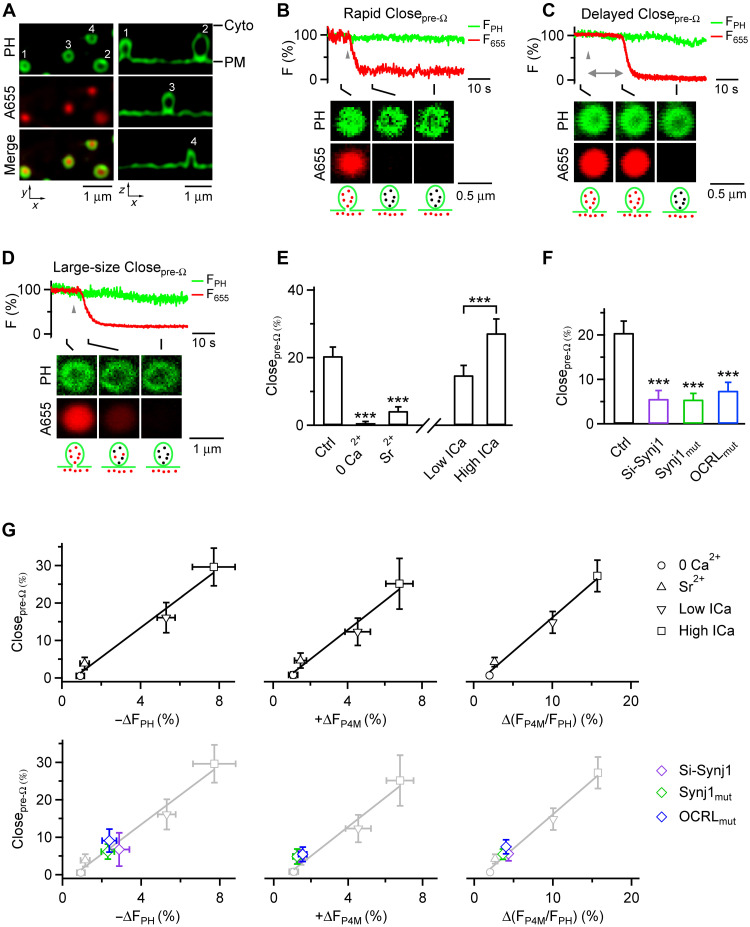
Synaptojanin-mediated PI(4,5)P_2_-to-PI4P transition triggers pre-Ω pore closure. (**A**) Preformed spots reflect mostly preformed Ω-profiles. Left: Confocal *XY*-plane images of PH_G_ (labeling PM) and A655 (bath) at the cell bottom showing 4 preformed PH_G_ rings/spots overlapping with A655 spots in resting conditions. Right: STED *XZ*-plane PH_G_ image through the center of preformed-spots on the left. Spots 1 to 3: Ω-shaped; spot 4: Λ-shaped. This image is reprinted from *10* with permission from Elsevier. (**B** to **D**) Pre-Ω pore closure (Close_pre-Ω_): F_PH_, F_655_, and confocal XY/Z_fix_ images show Close_pre-Ω_ at different times after depol_1s_ (triangle). Pore closure time is measured as the interval from depol_1s_ to the F_655_ decay onset (double arrow in C). Pore closure time in (C) is delayed compared to (B) or (D); the Ω-profile in (D) is larger than in (B) and (C). (**E**) Close_pre-Ω_ is calcium-dependent. Left: Close_pre-Ω_ percentage (mean + SEM) in Ctrl (33 cells), 0 Ca^2+^ (12 cells), or Sr^2+^ (12 cells). ***P < 0.001, ANOVA. Right: Control group divided into low ICa (<400 pA, *n* = 18 cells) and high ICa (>400 pA, *n* = 15 cells). ***P < 0.001, *t* test. (**F**) Close_pre-Ω_ percentage (mean + SEM) in control (33 cells), Si-Synj1 (16 cells), Synj1_mut_ (15 cells), or OCRL_mut_ (20 cells) after depol_1s_. ***P < 0.001, ANOVA. (**G**) Close_pre-Ω_ percentage plotted versus the PI(4,5)P_2_ decrease percentage (−ΔF_PH_, left), PI4P increase percentage (+ΔF_P4M_, middle), or the percentage increase of the ratio between F_P4M_ and F_PH_ [ΔF_P4M_/F_PH_ = F_P4M_/F_PH_ − 1; right]. Data were obtained from below. Upper: High ICa, low ICa, 0 Ca^2+^, and Sr^2+^. The lines are linear regression fit with a correlation coefficient of 0.95 (left), 0.98 (middle), and 0.99 (right), respectively. −ΔF_PH_ and +ΔF_P4M_ were taken from Fig. 1 (F and H); Close_pre-Ω_ from (E). Lower: Si-Synj1, Synj1_mut_, and OCRL_mut_; data in the upper panel are also shown in light gray. −ΔF_PH_ and +ΔF_P4M_ were taken from Fig. 1J; Close_pre-Ω_ from (F).

**Fig. 3.**
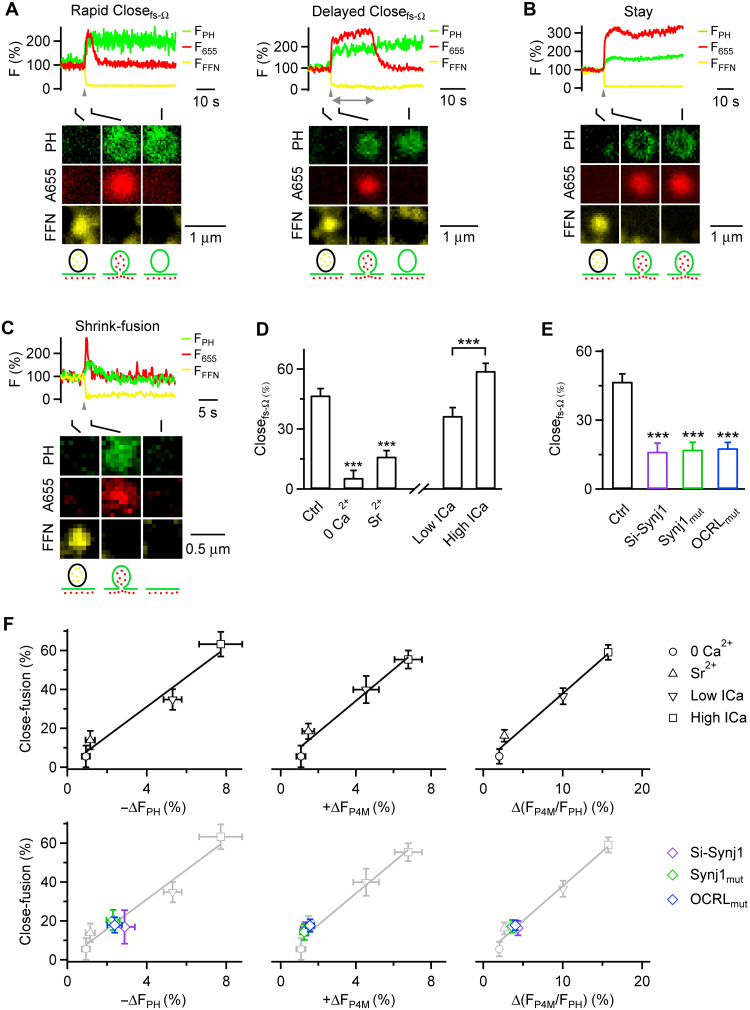
Synaptojanin-mediated PI(4,5)P_2_-to-PI4P transition triggers fs-Ω pore closure. (**A** to **C**) F_PH_, F_655_, F_FFN_, and confocal *XY*-plane images (at cell bottom) showing Close_fs-Ω_ (A: left, fast onset after depol_1s_; right, delayed onset after depol_1s_), stay fusion (B), and shrink fusion (C) (see also cartoon drawings at the bottom). The double arrow in (A) indicates fusion pore opening and closure time. Triangle: depol_1s_. (**D**) Close_fs-Ω_ is calcium-dependent. Left: depol_1s_-induced Close_fs-Ω_ percentage (mean + SEM) in Ctrl (33 cells), 0 Ca^2+^ (12 cells), or Sr^2+^ (12 cells). ***P < 0.001, ANOVA (comparing to Ctrl). Right: Similar plots as left, but with the control group divided into two subgroups, the low ICa (<400 pA, *n* = 18 cells) and high ICa group (>400 pA, *n* = 15 cells). ***P < 0.001, *t* test. (**E**) Synaptojanin is involved in mediating Close_fs-Ω_: the Close_fs-Ω_ percentage (mean + SEM) in Ctrl (33 cells), Si-Synj1 (16 cells), Synj1_mut_ (synaptojanin 1 R839C, 15 cells), or OCRL_mut_ (20 cells). Close_fs-Ω_ was measured after depol_1s_. ***P < 0.001, ANOVA (comparing to Ctrl). (**F**) Close_fs-Ω_ percentage plotted versus the PI4P increase percentage (+ΔF_P4M_, left), PI(4,5)P_2_ decrease percentage (−ΔF_PH_, middle), or the percent increase of the ratio between F_P4M_ and F_PH_ [ΔF_P4M_/F_PH_ = F_P4M_/F_PH_ − 1; right]. Data were obtained from a variety of conditions described below. Upper: High ICa, low ICa, 0 Ca^2+^, and Sr^2+^. The lines are linear regression fit with a correlation coefficient of 0.93 (left), 0.98 (middle), and 0.98 (right), respectively. −ΔF_PH_ and +ΔF_P4M_ were taken from Fig. 1 (F and H); Close_fs-Ω_ from (D). Lower: Si-Synj1, Synj1_mut_, and OCRL_mut_; data in the upper panel are also shown in light gray. −ΔF_PH_ and +ΔF_P4M_ were taken from Fig. 1J; Close_pre-Ω_ from (F).

**Fig. 4.**
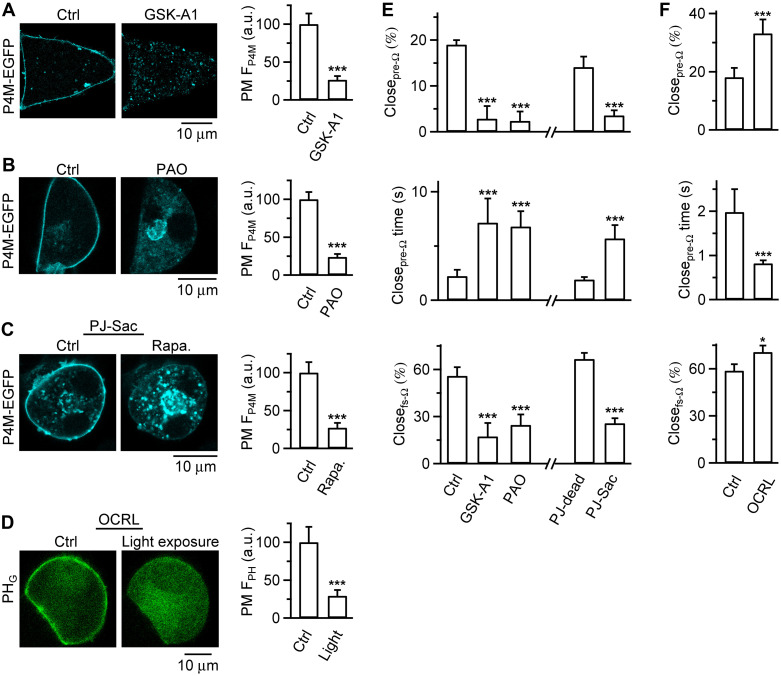
PI4P promotes while PI(4,5)P_2_ inhibits pore closure of both pre-Ω and fs-Ω. (**A**) Left: P4M-EGFP (PI4P indicator) images across the cell center before (Ctrl) and after GSK-A1 (100 nM, 5 min) in a chromaffin cell overexpressing P4M-EGFP. Right: P4M-EGFP fluorescence intensity at the plasma membrane (PM F_P4M_, mean + SEM) before (Ctrl) and after GSK-A1 (12 cells), normalized to the mean value in Ctrl (a.u., arbitrary unit). ***P < 0.001, *t* test. (**B**) Similar to (A), but before (Ctrl) and after PAO (20 μM, 10 min, 10 cells). (**C**) Similar to (A), but before (Ctrl) and after rapamycin (0.5 μM, 5 min) in PJ-Sac–transfected cells (10 cells). (**D**) Left: PH_G_ [PI(4,5)P_2_ indicator] images across the cell center before (Ctrl, left) and after blue light exposure (15 s) to a CIBN-CAAX/CRY2-OCRL and PH_G_ cotransfected cell. Right: PH_G_ fluorescence intensity at the PM (PM F_PH_, mean + SEM) before and after light exposure (10 cells), normalized to the mean value in Ctrl (a.u., arbitrary unit). ***P < 0.001, *t* test. (**E**) Close_pre-Ω_ percentage (top), pre-Ω pore closure time (Close_pre-Ω_ time; middle), and Close_fs-Ω_ percentage (bottom) in Ctrl (19 cells), GSK-A1 (17 cells), PAO (20 cells), or PJ-dead–transfected cells treated with rapamycin (36 cells, served as the control for PJ-Sac), or PJ-Sac–transfected cells treated with rapamycin (35 Cells), mean + SEM ***P < 0.001, ANOVA versus Ctrl or *t* test between PJ-dead and PJ-Sac. Close_pre-Ω_ and Close_fs-Ω_ were detected by imaging A655 and PH_G_ (except F). Close_pre-Ω_ time was measured as the interval from depol_1s_ onset to onset of F_655_ bleaching. (**F**) Same measurements as (E), in Ctrl (19 cells) or OCRL (CIBN-CAAX/CRY2-OCRL–transfected cells exposed to blue light, 19 cells), mean + SEM. *P < 0.05, *t* test; ***P < 0.001, *t* test. Close_pre-Ω_ and Close_fs-Ω_ were detected by imaging of A655 and P4M-EGFP (fig. S13).

**Fig. 5.**
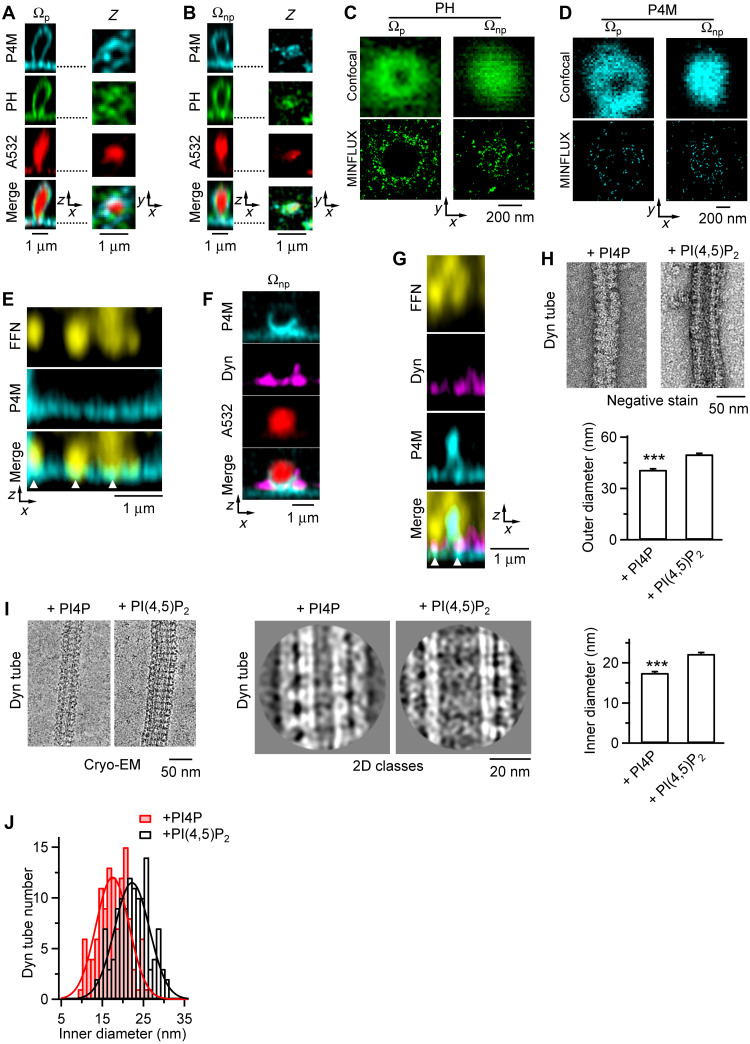
PI4P promotes dynamin to generate more constriction than PI(4,5)P_2_. (**A** and **B**) STED *XZ*-plane images of P4M-mTFP1 (labelling PI4P), PH_G_ [labelling PI(4,5)P_2_], and A532 (bath) for pre-Ω with a visible pore (Ω_p_, A) or a nonvisible pore (Ω_np_, B). *XY*-plane images across Ω’s pore region (dotted line) showed both PI4P and PI(4,5)P_2_ at the pore region. Similar observations were made in 29 cells (five cultures, each culture from two to four glands). (**C**) PI(4,5)P_2_ at the pore region: confocal (upper) and MINFLUX (lower) images of PH-SNAP-A647 (PH-domain attached with Alexa 647–tagged SNAP, labeling PI(4,5)P_2_) for a Ω_p_ and a Ω_np_ at the *XY* plane across the pore region. Imaging were performed on fixed cells. (**D**) PI4P at the pore region: similar to (C), except for P4M-SNAP-A647 (P4M attached with Alexa 647–tagged SNAP, labeling PI4P). (**E**) STED *XZ*-plane images of FFN206 (labeling vesicles) and P4M-mTFP1 (merged at bottom): Vesicles docked at PI4P clusters at PM (triangles). Similar observations were made in 26 cells (four cultures). (**F**) STED *XZ*-plane images of P4M-mTFP1, dynamin 2-mNeonGreen (Dyn), and A532 (merged at bottom): dynamin 2 and PI4P were at the pre-Ω’s pore region. Similar observations were made in 10 cells (two cultures). (**G**) STED *XZ*-plane images of FFN206, Dyn, and P4M-mTFP1 (merged at bottom): PI4P and dynamin 2 were colocalized at the vesicle docking site (triangles) ready for mediating fusion pore closure. Similar observations were made in 26 cells (four cultures). (**H**) Sampled negative stain images (upper) and the outer diameter (lower) of ∆PRD–dynamin 1 decorated 30% DOPS, 68% DOPC tubes (Dyn-tubes) in the presence of PI4P (2%, *n* = 50 tubes) or PI(4,5)P_2_ (2%, *n* = 50 tubes). ***P < 0.001, *t* test. (**I**) Sampled cryo-EM images (left), 2D class images (middle), and inner diameter (right) of Dyn-tubes in the presence of PI4P (2%, *n* = 120 tubes) or PI(4,5)P_2_ (2%, *n* = 120 tubes). ***P < 0.001, *t* test. (**J**) Distribution of Dyn-tube inner diameter in the presence of PI4P (2%, *n* = 120 tubes) or PI(4,5)P_2_ (2%, *n* = 120 tubes). Curves: gaussian fits.

**Fig. 6.**
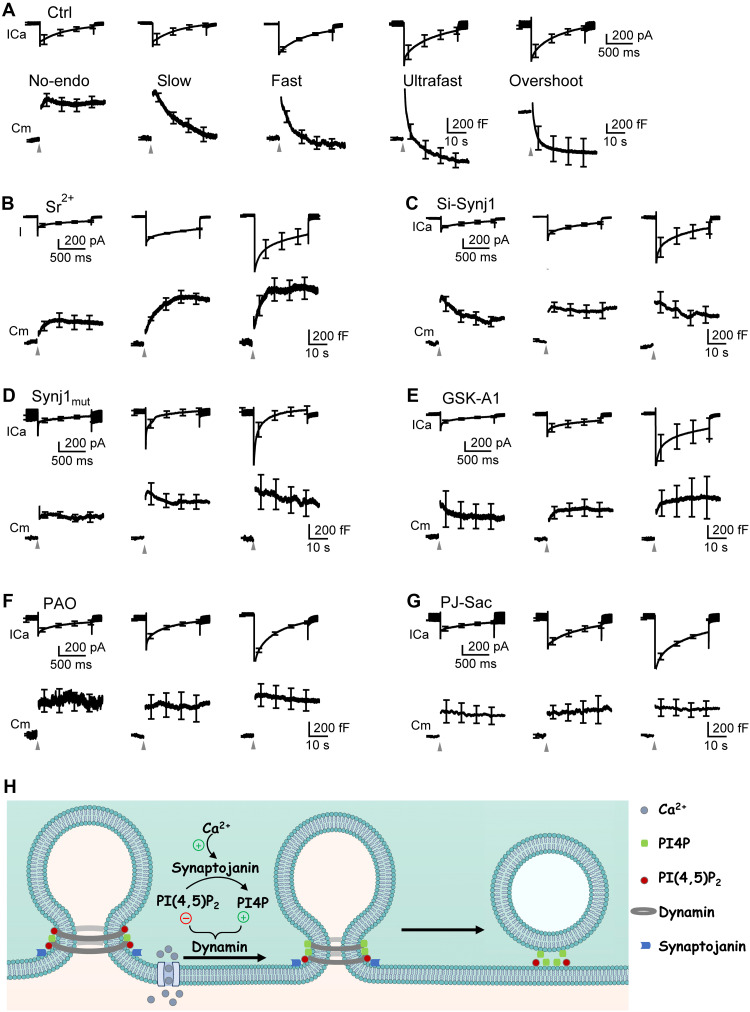
Synaptojanin-mediated PI(4,5)P_2_-to-PI4P transition and PI4P control multiple forms of calcium-dependent endocytosis. (**A**) Mean ICa (mean ± SEM, upper) and Cm (mean ± SEM, lower) induced by depol_1s_ (gray triangle) in five groups of chromaffin cells (from left to right): Group_no-endo_ (decay <30% ΔCm, seven cells), Group_slow_ (endocytic τ > 6 s, seven cells), Group_fast_ (τ: 0.6 to 6 s, seven cells), Group_ultrafast_ (τ < 0.6 s, seven cells), and Group_overshoot_ (decay >130% ΔCm, seven cells) in control chromaffin cells (Ctrl: 5 mM extracellular calcium). (**B**) Depol_1s_-induced strontium currents (mean ± SEM, upper) and Cm (mean ± SEM, lower) in cells with strontium current of 130 to 380 pA (six cells, left), 460 to 590 pA (five cells, middle), and 650 to 1300 pA (six cells, right). The extracellular calcium was replaced with strontium (Sr^2+^, 5 mM). (C to G) Depol_1s_-induced ICa (mean ± SEM, upper) and Cm (mean ± SEM, lower) in cells with ICa of 180 to 300 (left), 360 to 800 (middle), and 1000 to 1500 pA (right) in five conditions: (**C**) Si-Synj1 (left: five cells; middle: five cells; right: five cells); (**D**) Synj1_mut_ (left: five cells; middle: seven cells; right: five cells); (**E**) GSK-A1 (left: six cells; middle: five cells; right: six cells); (**F**) PAO (left: six cells; middle: eight cells; right: eight cells); (**G**) PJ-Sac (left: five cells; middle: five cells; right: eight cells). The ICa range covers the entire ICa range in control. (**H**) Schematic drawing of the model for initiation of the Ω-profile pore closure: calcium influx activates synaptojanin to convert PI(4,5)P_2_ into PI4P, relieving PI(4,5)P_2_ from inhibiting but enhancing PI4P to promote dynamin-mediated constriction and closure of the Ω-profile’s pore.

Our finding that depolarization-induced calcium influx triggers pore closure of pre-Ω and fs-Ω, as systematically characterized in adrenal chromaffin cells (*9*, *10*, *15*), raises the question of how calcium influx triggers pre-Ω/fs-Ω pore closure. We noticed that depolarization induces a rapid PI(4,5)P_2_ decrease and PI4P increase [PI(4,5)P_2_ decrease/PI4P increase] at the PM (see Fig. 1, A to D, described later), raising the possibility that depolarization-induced calcium influx generates the PI(4,5)P_2_ decrease/PI4P increase to trigger pre-Ω/fs-Ω pore closure. The present work examined this possibility systematically by determining whether calcium influx triggers the PI(4,5)P_2_ decrease/PI4P increase (Fig. 1); whether the PI(4,5)P_2_ decrease/PI4P increase reflects the PI(4,5)P_2_-to-PI4P transition mediated by a 5′-phosphatase, synaptojanin (Fig. 1); whether the PI(4,5)P_2_ decrease/PI4P increase triggers pre-Ω/fs-Ω pore closure (Figs. 2 and 3); whether PI4P and PI(4,5)P_2_ promote and inhibit pre-Ω/fs-Ω pore closure, respectively, through facilitation and inhibition of the key fission enzyme dynamin (Figs. 4 and 5); and whether the calcium-induced PI(4,5)P_2_ decrease/PI4P increase initiates diverse endocytic modes (Fig. 6). We also explored whether synaptojanin mutations associated with neurological disorders impair the PI(4,5)P_2_-to-PI4P transition, pre-Ω/fs-Ω pore closure, and endocytosis (Figs. 1 to 6).

### Depolarization induces rapid PI(4,5)P_2_ decrease/PI4P increase

Bovine adrenal chromaffin cells (primary culture) were overexpressed with phospholipase C delta PH domain attached with a fluorescent protein, such as mNeonGreen (PH_G_) or mCherry (PH_mCh_), for binding PI(4,5)P_2_ at the PM inner leaflet and thus detecting PI(4,5)P_2_ and labeling the PM at the cell bottom at the *XY* plane (Fig. 1, A to C; see also fig. S3A for an *XZ*-plane image) or the cell outline across the cell center at the *XY* plane (fig. S1A) (*10*, *15*, *16*). For the detection of PI4P, we overexpressed cells with a probe consisting of two tandem copies of P4M domain from *Legionella pneumophila* SidM fused to enhanced green fluorescent protein (EGFP; GFP-P4M-SidMx2, hereafter referred to as P4M-EGFP) (*17*). This 2xP4M configuration improves binding affinity and sensitivity for PI4P detection, allowing the probe to effectively label PI4P on the PM, visible at the cell bottom (Fig. 1, B and C) or the cell outline across the cell center (fig. S1B) (*17*). A 1-s depolarization (−80 to +10 mV, depol_1s_, whole-cell voltage clamp) induced calcium currents (ICa) and capacitance jump (ΔCm) and decays reflecting exo-endocytosis (Fig. 1A) (*9*, *16*). The depol_1s_ induced a rapid PH_G_ or PH_mCh_ fluorescence (F_PH_) decrease and P4M-EGFP fluorescence (F_P4M_) increase within milliseconds as detected at the cell-bottom at the *XY* plane (Fig. 1C) or the cell outline across the cell center (fig. S2), reflecting PI(4,5)P_2_ decrease and PI4P increase, respectively, at the PM. PI(4,5)P_2_ decrease paralleled PI4P increase, as concurrently recorded from the same cells (Fig. 1, C and D).

The depol_1s_-induced PI(4,5)P_2_ decrease (PH_G_ or PH_mCh_ decrease) was similar with or without overexpression of P4M-EGFP to detect PI4P (42 cells), suggesting that P4M-EGFP overexpression does not affect the observed PI(4,5)P_2_ change; likewise, the depol_1s_-induced PI4P increase (P4M-EGFP increase) was similar with or without overexpression of PH_G_ (or PH_mCh_) overexpression (36 cells), suggesting that overexpression of PH_G_ or PH_mCh_ does not affect the observed PI4P change. The PI(4,5)P_2_ decrease and PI4P increase measured with confocal imaging were relatively small (Fig. 1, C and D), likely because of the confocal resolution. Supporting this possibility, stimulated emission depletion (STED) microscopy showed a much larger PI(4,5)P_2_ decrease (37 ± 2% decrease, 55 cells; range, 5 to 76%; fig. S3).

We verified the P4M-EGFP-detected PI4P signal using an independent and structurally distinct PI4P probe, oxysterol-binding protein attached with EGFP (OSBP-EGFP), which binds PI4P via its PH domain (*18*). The overexpressed OSBP-EGFP exhibited spatial and temporal dynamics similar to those of P4M-EGFP following depol_1s_ (fig. S4), strengthening our conclusion that depol_1s_-induced P4M-EGFP increase reflects PI4P increase, rather than other phosphoinositides (*19*, *20*).

### Calcium influx activates the 5′-phosphatase synaptojanin to convert PI(4,5)P_2_ to PI4P

Both PI(4,5)P_2_ decrease and PI4P increase were triggered by depolarization-induced calcium influx, because (i) removing the extracellular calcium (0 Ca^2+^) blocked both F_PH_ decrease and F_P4M_ increase (Fig. 1, E and F); (ii) replacing the extracellular calcium with strontium (Sr^2+^) largely inhibited both F_PH_ decrease and F_P4M_ increase, as obtained from *XY*-plane imaging (Fig. 1, E and F) or *XZ*-plane imaging (fig. S3); and (iii) both F_PH_ decrease and F_P4M_ increase were much larger in cells with higher ICa (high ICa, > 400 pA) than with lower ICa (low ICa, < 400 pA), as obtained from *XY*-plane imaging (Fig. 1, G and H) or *XZ*-plane imaging (fig. S3).

The parallel time course and the calcium dependence of PI(4,5)P_2_ decrease and PI4P increase imply that calcium influx induces the PI(4,5)P_2_-to-PI4P transition by activating 5′-phosphatase to dephosphorylate PI(4,5)P_2_. Three sets of evidence further support this implication. First, transfection of synaptojanin 1 Si-RNA (Si-Synj1) in chromaffin cells reduced synaptojanin 1 expression (fig. S5) without affecting ICa (Fig. 1I) but inhibited both PI(4,5)P_2_ decrease and PI4P increase (Fig. 1J). This result suggests that synaptojanin 1 mediates rapid PI(4,5)P_2_-to-PI4P transition, resulting in rapid concurrent PI(4,5)P_2_ decrease and PI4P increase after depol_1s_. Second, we transfected chromaffin cells with synaptojanin 1 R839C, a synaptojanin 1 dominant-negative mutant (Synj1_mut_) with impaired dephosphorylation function (*21*) and clinically associated with early-onset Parkinsonism and seizure (*22*). This transfection did not affect ICa (Fig. 1I), but substantially reduced depol_1s_-induced PI(4,5)P_2_ decrease and PI4P increase (Fig. 1J). Third, we transfected cells with a mutant oculocerebrorenal syndrome of Lowe’s (OCRL) inositol polyphosphate-5-phosphatase domain (OCRL_mut_, D523G), which is catalytically inactive but can still bind PI(4,5)P_2_ and acts as a dominant negative by competing with and thus inhibiting endogenous 5′-phosphatases (*23*). To acutely bring this mutant to the PM, we used an optogenetic approach using light-induced dimerization between CIBN-CAAX (amino acids 1 to 170 of *Arabidopsis thaliana* CIB1 attached with a CAAX motif) localized at the PM and CRY2 (cryptochrome 2) attached with the OCRL mutant (CRY2-OCRL_mut_; see fig. S6 for a schematic drawing of this approach) (*23*). Light exposure to CIBN-CAAX/CRY2-OCRL_mut_–transfected cells led to inhibition of depol_1s_-induced PI(4,5)P_2_ decrease and PI4P increase (Fig. 1J) without affecting ICa (Fig. 1I) or the baseline PH_G_ fluorescence before depol_1s_ (fig. S6). These results suggest that depolarization-induced calcium influx activates synaptojanin 1 to dephosphorylate PI(4,5)P_2_ into PI4P, rapidly increasing PI4P at the PM.

### PI(4,5)P_2_-to-PI4P transition triggers preformed Ω-profile pore closure

Knowing that calcium influx activates synaptojanin 1 to mediate the PI(4,5)P_2_-to-PI4P transition (Fig. 1), we tested the hypothesis that the calcium influx induces the PI(4,5)P_2_-to-PI4P transition to trigger pore closure of pre-Ω (Close_pre-Ω_, this section, Fig. 2) and fs-Ω (Close_fs-Ω_, next section, Fig. 3). To test this hypothesis, we first outline the method for measuring pre-Ω closure in this section. We will then investigate three key points: (i) whether inhibiting calcium influx reduces the PI(4,5)P_2_-to-PI4P transition and Close_pre-Ω_ proportionally; (ii) whether inhibiting the PI(4,5)P_2_-to-PI4P transition, without inhibiting calcium influx, reduces Close_pre-Ω_; and (iii) whether the reduction in the PI(4,5)P_2_-to-PI4P transition is sufficient to account for the decrease of Close_pre-Ω_ caused by inhibition of calcium influx.

To detect Close_pre-Ω_, we overexpressed chromaffin cells with PH_G_ to label the PM, including the pre-Ω’s membrane, and applied Atto 655 (A655), a membrane-impermeant fluorescent dye, to the bath solution. This allowed A655 in the bath solution to diffuse through the pore into the Ω-profile, thereby labeling the pre-Ω structure at the PM (Figs. 1A and 2A, also see Materials and Methods) (*10*, *15*, *16*). Confocal or STED *XY*-plane imaging (near cell bottom) showed preformed PH_G_ spots (or rings) overlapped with A655 spots in resting conditions (preformed spots), reflecting mostly (88 ± 3%, 13 cells) preformed Ω-profiles (pre-Ω) observed at the *XZ* plane, and occasionally preformed Λ-shape profiles (e.g., Fig. 2A) (*10*). Depol_1s_-induced ICa triggered preformed-spot pore closure (Fig. 2, B to D), detected as A655 fluorescence (strongly excited) dimming, while PH_G_ fluorescence (F_PH_, weakly excited) sustained or dimmed with a delay, due to pore closure that prevented bleached A655 (by strong excitation) from exchanging with bath fluorescent A655 (*9*, *10*, *15*, *16*). Preformed-spot pore closure, termed Close_pre-Ω_, reflects nearly entirely pre-Ω pore closure because (i) preformed-spots are mostly pre-Ω (*10*), (ii) preformed Λ-shape profiles rarely proceed to pore closure compared to pre-Ω (*10*), and (iii) pre-Ω pore closure, which forms vesicles of ~200 to 1500 nm in diameter, has been directly observed with STED *XZ*-plane imaging (e.g., fig. S7) (*10*). Close_pre-Ω_ may generate vesicles much larger than dense-core vesicles (e.g., Fig. 2D) and thus may contribute to the generation of bulk endocytosis (*10*).

Although Close_pre-Ω_ (or Close_fs-Ω_, next section) was measured with overexpressed PH_G_, we have repeatedly shown that PH_G_ overexpression does not affect endocytosis, Close_pre-Ω_, or Close_fs-Ω_ in chromaffin cells, because similar endocytosis, Close_pre-Ω_, and fusion pore closure were observed with imaging of A655 alone (*10*), or imaging of A655 and Alexa 488 (*9*, *16*). Further supporting this observation, we showed in the present work that Close_pre-Ω_ and Close_fs-Ω_ detected with imaging of PH_G_ and A655 were similar to those detected with imaging of P4M-EGFP and A655 [described later in Fig. 4, E (Ctrl) and F (Ctrl), and fig. S13].

Three manipulations of calcium influx described in Fig. 1, including 0 Ca^2+^, Sr^2+^, and low versus high ICa, not only inhibited the PI(4,5)P_2_-to-PI4P transition (Fig. 1, F and H) but also abolished or substantially reduced Close_pre-Ω_ (Fig. 2E), consistent with our recent finding that calcium influx triggers Close_pre-Ω_ (*10*). The Close_pre-Ω_ percentages obtained in these conditions (Fig. 2E) were plotted versus the corresponding PI(4,5)P_2_ decrease (obtained from Fig. 1, F and H), PI4P increase (obtained from Fig. 1, F and H), or the ratio between PI4P and PI(4,5)P_2_. All three plots were fitted well with a linear regression line (Fig. 2G, upper), supporting our suggestion that calcium influx triggers Close_pre-Ω_ by inducing the PI(4,5)P_2_-to-PI4P transition.

Three approaches described above that inhibit 5′-phosphotase but not ICa (Fig. 1, I and J), including Si-Synj1, synaptojanin 1 R839C mutant overexpression, and the optogenetic approach with OCRL_mut_ transfection, not only inhibited PI(4,5)P_2_-to-PI4P transition (Fig. 1J) but also substantially reduced Close_pre-Ω_ (Fig. 2F), strengthening our suggestion that the PI(4,5)P_2_-to-PI4P transition triggers Close_pre-Ω_. Data quantification showed that these three approaches reduced the PI(4,5)P_2_ decrease amplitude from ~6.5 to ~2.3 to 2.8% (Fig. 1J), the PI4P increase amplitude from ~6.3 to ~1.2 to 1.5% (Fig. 1J), and the Close_pre-Ω_ percentage from ~20.0 to ~5.4 to 7.4% (Fig. 2F).

The resulting Close_pre-Ω_ percentage (~5.4 to 7.4%) was in the same range as that (2.1 to 8.9%) predicted from the linear regression fit obtained with calcium manipulations but using the PI(4,5)P_2_ decrease (2.3 to 2.8%) and PI4P increase (1.2 to 1.5%) values obtained with synaptojanin manipulations (Fig. 2G, lower; *P* > 0.1, *t* test). These three synaptojanin manipulations also reduced the ratio between PI4P and PI(4,5)P_2_, which was also similar to that predicted from the linear regression fit obtained with calcium manipulations (Fig. 2G, lower). The similarity between the Close_pre-Ω_ measured after 5′-phosphotase inhibition and that predicted from calcium manipulation (Fig. 2G) strengthens the suggestion that calcium influx triggers Close_pre-Ω_ by inducing the PI(4,5)P_2_-to-PI4P transition.

### PI(4,5)P_2_-to-PI4P transition triggers fusion pore closure

To determine whether calcium influx also triggers Close_fs-Ω_ by inducing the PI(4,5)P_2_-to-PI4P transition, we detected vesicle fusion by preloading and thus labeling the vesicles with fluorescent false neurotransmitter FFN511 (see Materials and Methods) (*10*, *24*, *25*). FFN511 spots reflecting individual vesicles were observed at the confocal microscope’s *XY* plane with a *Z*-axis focus near the cell bottom [Figs. 1A, schematics, and 3, A to C; see also (*10*) and (*25*)].

We detected vesicle fusion as FFN511 spot fluorescence (F_FFN_) decay that reflects FFN511 release, accompanied by PH_G_ and A655 spot appearance due to PH_G_ and A655 diffusion from the PM and the bath into the fs-Ω, respectively (Fig. 3, A to C) (*9*, *10*, *16*, *24*, *25*). PH_G_/A655 spots may reflect fs-Ω undergoing pore closure (termed Close_fs-Ω_, close fusion, or kiss-and-run, Fig. 3A), unchanged (stay fusion, Fig. 3B), or fs-Ω shrinking (shrink fusion, Fig. 3C), as verified with STED *XZ*-plane imaging (e.g., fig. S8) (*10*, *15*, *26*). Analogous to Close_pre-Ω_, Close_fs-Ω_ was detected as F_655_ dimming, while F_PH_ sustained or decayed with a delay (Fig. 3A and fig. S8A) (*10*, *15*, *26*).

Three manipulations of calcium influx described above (Fig. 1E), including 0 Ca^2+^, Sr^2+^, and low versus high ICa, not only inhibited PI(4,5)P_2_-to-PI4P transition (Fig. 1H) but also abolished or substantially reduced Close_fs-Ω_ (Fig. 3D), confirming the previous finding that calcium influx triggers fs-Ω pore closure (*9*, *15*, *16*). The Close_fs-Ω_ percentages obtained in these conditions (Fig. 3D) were plotted versus the corresponding PI(4,5)P_2_ decrease (obtained from Fig. 1, F and H), PI4P increase (obtained from Fig. 1, F and H), or the ratio between PI4P and PI(4,5)P_2_. All three plots were fitted well with a linear regression line (Fig. 3F, upper), supporting our suggestion that calcium influx triggers Close_fs-Ω_ by inducing the PI(4,5)P_2_-to-PI4P transition.

Three approaches described above that inhibit 5′-phosphotase but not ICa (Fig. 1, I and J), including Si-Synj1, synaptojanin 1 R839C mutant overexpression, and the optogenetic approach with OCRL_mut_ transfection, not only inhibited PI(4,5)P_2_-to-PI4P transition (Fig. 1J) but also substantially reduced Close_fs-Ω_ (Fig. 3E), suggesting that PI(4,5)P_2_-to-PI4P transition triggers Close_fs-Ω_. More specifically, these three approaches reduced the PI(4,5)P_2_ decrease amplitude from ~6.5 to ~2.3 to 2.8% (Fig. 1J), the PI4P increase amplitude from ~6.3 to ~1.2 to 1.5% (Fig. 1J), and the Close_fs-Ω_ percentage from ~46 to ~16.2 to 17.7% (Fig. 3E). The resulting Close_fs-Ω_ percentage (~16.2 to 17.7%) was similar to that (12.0 to 18.7%) predicted from the linear regression fit obtained with calcium manipulations but using the PI(4,5)P_2_ decrease (2.3 to 2.8%) and PI4P increase (1.2 to 1.5%) values obtained with synaptojanin manipulations (Fig. 3F, lower; *P* > 0.1, *t* test). These three manipulations also reduced the ratio between PI4P and PI(4,5)P_2_, which was also similar to that predicted from the linear regression fit obtained with calcium manipulations (Fig. 3F, lower). The similarity between the Close_fs-Ω_ measured after 5′-phosphotase inhibition and that predicted from calcium manipulations (Fig. 3F) strengthens the suggestion that calcium influx triggers fs-Ω pore closure by inducing the PI(4,5)P_2_-to-PI4P transition.

### PI4P is essential for pre-Ω and fs-Ω pore closure

Our finding that PI(4,5)P_2_-to-PI4P transition triggers pre-Ω/fs-Ω closure (Figs. 2 and 3) implies that PI4P may facilitate pre-Ω/fs-Ω closure and/or that PI(4,5)P_2_ may inhibit pre-Ω/fs-Ω closure. We distinguished these possibilities by determining whether reducing PI4P (Fig. 4, A to C) or PI(4,5)P_2_ (Fig. 4D) influences Close_pre-Ω_ and Close_fs-Ω_ (Fig. 4, E and F).

Three sets of evidence suggest that PI4P is essential for Close_pre-Ω_. First, GSK-A1, a potent and highly specific inhibitor of the phosphatidylinositol (PI) 4 kinase (PI4K) that mediates the PI-to-PI4P transition (*27*, *28*), reduced P4M-EGFP-detected PI4P at the PM (Fig. 4A and fig. S9A) but not PH_G_-detected PI(4,5)P_2_ at the PM (fig. S9, B and C). GSK-A1 substantially reduced depol_1s_-induced Close_pre-Ω_ (Fig. 4E) and prolonged the closure time (Fig. 4E), the time between depol_1s_ and the onset of the F_655_ decay (e.g., Fig. 2C, double arrow) (*10*). Second, similar to GSK-A1, another PI4K inhibitor, phenylarsine oxide (PAO) (*17*, *29*), reduced P4M-EGFP–detected PI4P (Fig. 4B and fig. S9D) but not PH_G_-detected PI(4,5)P_2_ at the PM (fig. S9, E and F); PAO substantially reduced depol_1s_-induced Close_pre-Ω_ and prolonged the closure time (Fig. 4E).

Third, we used a well-established chemical-genetic strategy that selectively and acutely reduces PI4P at the PM (*29*). This strategy used rapamycin-inducible dimerization between FKBP (FK506 binding protein 12) and FRB domains (fragment of mTOR that binds rapamycin, anchored at the PM) to recruit enzymes to the PM (fig. S10A); the enzyme was a chimera of the *Saccharomyces cerevisiae* sac1 phosphatase, which dephosphorylates PI4P into PI, and an inactive inositol polyphosphate-5-phosphatase E (INPP5E) domain; this system was named PJ-Sac (PJ refers to Pseudojanin, fig. S10, A and B) (*29*). PJ-Sac specifically reduced PI4P, but not PI(4,5)P_2_ at the PM; an inactive mutant sac1 phosphatase was generated as the control, named PJ-dead (fig. S10A), which does not affect PI4P or PI(4,5)P_2_ levels (*29*). Compared to PJ-dead–transfected cells, rapamycin application to PJ-Sac–transfected cells within minutes reduced P4M-EGFP–detected PI4P but not PH_G_-detected PI(4,5)P_2_ at the PM (Fig. 4C and figs. S10, C to E, and S11), blocked depol_1s_-induced Close_pre-Ω_, and prolonged the pore closure time (Fig. 4E).

The above three sets of evidence suggest that PI4P is required for pre-Ω pore closure. Similar to Close_pre-Ω_, decreasing PI4P by application of GSK-A1 or PAO or rapamycin application to PJ-Sac–transfected cells substantially inhibited Close_fs-Ω_ (Fig. 4E), suggesting that PI4P is also required for fusion pore closure or kiss-and-run fusion.

### PI(4,5)P_2_ inhibits pre-Ω and fs-Ω pore closure

To determine whether PI(4,5)P_2_ is essential for pre-Ω/fs-Ω pore closure, we reduced PI(4,5)P_2_ with the optogenetic approach using light-induced dimerization between CIBN-CAAX and CRY2 attached to OCRL’s inositol polyphosphate-5-phosphatase domain (CRY2-OCRL) to dephosphorylate PI(4,5)P_2_ (*23*). Blue light exposure to CIBN-CAAX/CRY2-OCRL–transfected cells reduced PH_G_-detected PI(4,5)P_2_ at the PM (Fig. 4D and fig. S12A) (*23*), depol_1s_-induced fusion spots observed at the cell-bottom membrane (fig. S12B), and depol_1s_-induced capacitance jump (fig. S12C), but not ICa (fig. S12C). These results are consistent with the reported exocytic roles of PI(4,5)P_2_ (*30*). Furthermore, this optogenetic approach increased depol_1s_-induced Close_pre-Ω_ and Close_fs-Ω_ (Fig. 4F and fig. S13) and shortened their close time (Fig. 4F), the time between the onset of fusion and F_655_ bleaching (e.g., Fig. 3A, double arrow) (*10*, *15*). Thus, unlike PI4P, PI(4,5)P_2_ is not essential for pre-Ω/fs-Ω pore closure; instead, it inhibits pre-Ω/fs-Ω pore closure.

For the PI(4,5)P2 reduction experiments, we detected Close_pre-Ω_ and Close_fs-Ω_ by imaging A655 and P4M-EGFP (overexpressed, see fig. S13), rather than A655 and PH_G_. In control conditions, Close_pre-Ω_ and Close_fs-Ω_ detected with A655/P4M-EGFP imaging (Fig. 4E, Ctrl) were similar to those with A655/PH_G_ imaging (Fig. 4F, Ctrl), suggesting that overexpressing PH_G_ or P4M-EGFP does not affect Close_pre-Ω_ or Close_fs-Ω_ measured in chromaffin cells.

### PI4P promotes dynamin for more efficient constriction than PI(4,5)P_2_

Since dynamin mediates pre-Ω/fs-Ω pore closure in chromaffin cells (*15*, *16*), our finding that PI4P promotes while PI(4,5)P_2_ inhibits Close_pre-Ω_/Close_fs-Ω_ suggests an opposing regulatory mechanism where these two phosphoinositides exert counterbalancing control over dynamin function. Two sets of evidence further support this suggestion (Fig. 5, A to J). First, PH_G_-labeled PI(4,5)P_2_ and P4M-mTFP1–labeled PI4P were present at the same pre-Ω pore region (Fig. 5, A and B) and the FFN206-labeled vesicle docking site at the PM (Fig. 5E and fig. S14, 29 cells). MINFLUX imaging with ~3 nm localization precision (*31*) revealed individual soluble *N*-ethylmaleimide–sensitive factor attachment protein (SNAP) tag–labeled PI(4,5)P_2_ and PI4P molecules densely packed at the pore region [PI(4,5)P_2_: 24 pores and eight cells; PI4P: 27 pores and eight cells; Fig. 5, C and D]. P4M-mTFP1 fluorescence was higher at the neck region of the pre-Ω (18 pre-Ω and four cells; fig. S15), suggesting a higher PI4P concentration at the neck region. Similarly, dynamin 2–mNeonGreen was localized to the same regions, either at the P4M-mTFP1–labeled pre-Ω pore region or FFN206-labeled vesicle docking site at the PM (26 cells; Fig. 5, F and G). Thus, PI(4,5)P_2_ and PI4P may physically interact with dynamin at pre-Ω/fs-Ω’s pore region. Second, 1-hour incubation of purified dynamin 1 with DOPS (1,2-dioleoyl-*sn*-glycero-3-phospho-l-serine) liposomes containing PI4P or PI(4,5)P_2_ generated dynamin-coated nanotubes, as observed with electron microscopy (EM; Fig. 5, H to J). With negative staining, dynamin-decorated, PI4P-containing nanotubes’ outer diameter (40.9 ± 0.6 nm, *n* = 50) was significantly smaller than PI(4,5)P_2_-containing nanotubes (50.0 ± 0.6 nm, *n* = 50; Fig. 5H), suggesting that PI4P promotes more dynamin-mediated constriction than PI(4,5)P_2_. To measure the inner diameter (difficult to measure with negative staining), we performed cryo-EM: dynamin-decorated, PI4P-containing nanotube’s inner diameter (17.5 ± 0.3 nm, *n* = 120) was substantially smaller than PI(4,5)P_2_-containing nanotube (22.2 ± 0.4 nm, *n* = 120; Fig. 5, I and J). The smallest inner diameter reached ~10 nm with PI4P, but could not reach ~15 nm with PI(4,5)P_2_ (Fig. 5J). Thus, PI4P-dynamin interaction produces more constriction than PI(4,5)P_2_-dynamin interaction, consistent with our live-cell data that PI4P, but not PI(4,5)P_2_, is essential for pore closure.

### PI(4,5)P_2_-to-PI4P transition and PI4P are essential for diverse modes of endocytosis

We have previously found that calcium influx initiates diverse modes of endocytosis primarily by triggering pre-Ω/fs-Ω pore closure (*10*, *15*). Together with this finding (*10*, *15*), our finding that calcium influx induces the PI(4,5)P_2_-to-PI4P transition to trigger pre-Ω/fs-Ω pore closure (Figs. 1 to 3) suggests that calcium influx initiates diverse modes of endocytosis largely by inducing the PI(4,5)P_2_-to-PI4P transition. Here, we verified this suggestion by determining how reducing calcium influx, PI(4,5)P_2_-to-PI4P transition or PI4P affects five modes of calcium-triggered endocytosis observed with whole-cell capacitance recordings.

Five distinct modes of endocytosis were previously characterized systematically when chromaffin cells were divided into five groups based on the decay of the whole-cell capacitance (Cm) after the jump induced by depol_1s_: (i) no endocytosis (Group_no-endo_, decay <30% ΔCm), (ii) slow endocytosis (Group_slow_, endocytic τ > 6 s), (iii) fast endocytosis (Group_fast_, τ: 0.6 to 6 s), (iv) ultrafast endocytosis (Group_ultrafast_, τ < 0.6 s), and (v) overshoot endocytosis [Group_overshoot_, decay > 130% ΔCm, Fig. 6A; see (*10*) for detail]. Calcium influx triggers endocytosis in each of these five groups: larger ICa induces faster and larger amplitude of endocytosis (Fig. 6A), whereas strontium abolishes endocytosis (see (*10*) for detail).

Replacing the extracellular calcium with strontium resulted in the block of the Cm decay, the endocytosis summed from the entire cell, regardless of the strontium current amplitude (Fig. 6B), confirming that calcium influx triggers each of the five endocytic modes as previously characterized (*10*). Inhibition of the PI(4,5)P_2_-to-PI4P transition by Si-Synj1 or transfection of the dominant-negative mutant synaptojanin 1 R839C inhibited the Cm decay–indicated endocytosis (Fig. 6, C and D). The inhibition was observed in all three cell groups divided on the basis of the ICa amplitude (Fig. 6, C and D), indicating inhibition of endocytosis throughout the entire ICa range that generates five distinct endocytic modes in control. These results suggest that PI(4,5)P_2_-to-PI4P transition is essential for diverse endocytic modes, including slow, fast, ultrafast, and overshoot endocytosis observed in control. Similarly, reducing PI4P level at the PM by application of GSK-A1 or PAO or rapamycin application to PJ-Sac–transfected cells substantially inhibited Cm decay–indicated endocytosis in three cell groups with different ICa (Fig. 6, E to G), indicating inhibition of endocytosis throughout the entire ICa range that generates five distinct endocytic modes in control. These results suggest that PI4P is required for mediating these five endocytic modes. Together, block of calcium influx, synaptojanin 1–mediated PI(4,5)P_2_-to-PI4P transition, or PI4P inhibits Cm decay–indicated endocytosis (Fig. 6, A to G), further supporting our suggestion that calcium influx initiates diverse endocytic modes, including slow, fast, ultrafast, and overshoot endocytosis, by inducing synaptojanin 1–mediated PI(4,5)P_2_-to-PI4P transition to trigger pre-Ω/fs-Ω closure.

## DISCUSSION

### A model for the initiation of fission, endocytosis, and kiss-and-run fusion

We demonstrated that calcium influx induces the rapid conversion of PI(4,5)P_2_ to PI4P at the PM within milliseconds by activating the 5′ phosphatase synaptojanin (Fig. 1). This calcium-driven PI(4,5)P_2_-to-PI4P transition triggers the pre-Ω and fs-Ω pore closure (Figs. 2 and 3), which underlie various endocytic modes, including slow, fast, ultrafast, overshoot, and bulk (large vesicle formation) endocytosis, as well as the kiss-and-run fusion (*10*). We showed that PI4P is essential for catalyzing pre-Ω/fs-Ω pore closure, while PI(4,5)P_2_ inhibits this process (Fig. 4), suggesting that the PI(4,5)P_2_-to-PI4P transition promotes pore closure by increasing PI4P levels while decreasing PI(4,5)P_2_. PI4P more effectively facilitates dynamin-mediated nanotube constriction than PI(4,5)P_2_ (Fig. 5), offering an explanation for how the PI(4,5)P_2_-to-PI4P transition triggers pre-Ω/fs-Ω pore closure by altering the PI4P-to-PI(4,5)P_2_ ratio to promote dynamin activity. Reducing calcium influx, inhibiting synaptojanin-mediated PI(4,5)P_2_-to-PI4P conversion, or lowering PI4P levels at the PM all impaired various endocytic modes, including slow, fast, ultrafast, and overshoot endocytosis (Fig. 6), all of which are primarily driven by calcium-induced pre-Ω/fs-Ω pore closure (*10**,* *15*). Together, these results delineate a rapid metabolic pathway in initiating fission and endocytosis (Fig. 6H): depolarization-induced calcium influx activates synaptojanin to catalyze the PI(4,5)P_2_-to-PI4P conversion, which increases the PI4P-to-PI(4,5)P_2_ ratio, promoting dynamin-mediated closure of pre-Ω/fs-Ω pores, thereby generating diverse modes of endocytosis and kiss-and-run fusion. This pathway may have a profound and broad impact on the study of endocytosis, membrane fission, exocytosis, and disorders related to impaired phosphoinositide metabolism, as discussed below.

### Broad impacts in endocytosis, fusion, and exo-endocytosis coupling

In addition to triggering exocytosis, calcium influx is known to trigger various endocytic modes (slow, fast, ultrafast, overshoot, and bulk endocytosis) and kiss-and-run in endocrine cells and neurons (*9*, *10*, *15*, *32*–*42*) [but see (*43*)]. Calcium influx is thus considered to couple endocytosis to exocytosis; increased calcium influx triggers not only greater exocytosis but also faster and more extensive endocytosis to recycle vesicles and maintain homeostasis of the PM (*4*, *33*, *34*). Given the involvement of numerous proteins and lipids in the flat-to-round endocytic curvature transition (*1*–*6*), how calcium influx coordinates these molecules to generate endocytic vesicles and thereby couple endocytosis to exocytosis appears highly challenging to address (*4*, *8*). The present work proposes a phosphoinositide metabolic pathway (Fig. 6H) that translates the physiological demand for recycling exocytosed vesicles into the initiation of endocytosis. This pathway couples endocytosis to exocytosis by specifically controlling the final step of vesicle formation, the dynamin-mediated closure of the pre-Ω/fs-Ω pore. This pathway strategically uses pre-Ω made before exocytosis or fs-Ω made during exocytosis to generate vesicles, avoiding the need to generate vesicles from flat membranes that may involve orchestrating tens of different molecules to generate large curvature transitions. As a result, this strategy allows for the high speed and capacity of vesicle retrieval by “simply” closing the pre-Ω/fs-Ω’s pore. The overall contribution of pre-Ω closure versus fs-Ω closure is about half each but varies depending on the mode of endocytosis (*10*), which is consistent with, and may explain, the nonidentical vesicular proteins being retrieved versus being exocytosed at synapses (*44*–*46*). Larger calcium influxes not only stimulate increased exocytosis but also induces more PI(4,5)P_2_-to-PI4P transition (Fig. 1, E to H), initiating more dynamin-mediated pre-Ω/fs-Ω closure (Figs. 2, E to G, and 3, D to F), leading to enhanced and accelerated endocytosis and kiss-and-run fusion to recycle vesicles (*10*).

### Mechanistic insight into diseases associated with phosphoinositide metabolism

Impairment of the endocytosis initiation pathway revealed here (Fig. 6H) may contribute to the development of disorders associated with impaired phosphoinositide metabolism. Numerous synaptojanin 1 mutations with compromised phosphatase activity, such as R258Q, R459P, R839C, Y888C, and Q647R, have been associated with Parkinsonism, seizures, severe intellectual disabilities, and neurodegeneration (*11*, *12*). We found that synaptojanin 1 R839C, a synaptojanin 1 dominant-negative mutant with impaired dephosphorylation function (*21*) and clinically associated with early-onset Parkinsonism and seizure (*22*), impaired the PI(4,5)P_2_-to-PI4P transition (Fig. 1), pre-Ω/fs-Ω pore closures (Figs. 2 and 3), diverse endocytic modes (slow, fast, ultrafast, and overshoot; Fig. 6), and kiss-and-run fusion (Fig. 3). These findings provide the molecular and cellular mechanism that may contribute to the generation of early-onset Parkinsonism and seizure.

In addition to synaptojanin 1 mutations associated with neurological disorders, mutations in PIP5K1C (PIPK1γ), which phosphorylates PI4P to generate PI(4,5)P_2_, are associated with lethal congenital contractural syndrome type 3 (*47*); mutations in OCRL, the inositol 5-phosphatase responsible for converting PI(4,5)P_2_ into PI4P, result in Lowe syndrome (*13*); mutations in PI4KA that synthesizes PI4P have been linked to polymicrogyria (*14*). Notably, fibroblasts derived from Lowe syndrome patients display an endocytic defect and a marked increase in U-shaped clathrin-coated pits (*13*). This observation provides compelling evidence for our model: when PI(4,5)P_2_-to-PI4P conversion fails, dynamin-mediated membrane constriction is impaired, resulting in these characteristic U-shaped structures rather than the constricted Ω-shaped pits (*13*). This notable correlation between our molecular findings and the observed cellular phenotypes in Lowe syndrome (*13*) underscores the critical role of phosphoinositide conversion in facilitating dynamin-mediated pore closure during endocytosis, directly connecting defects in phosphoinositide metabolism in patient cells to impaired vesicle recycling and consequent neurological dysfunction of the disease.

### Phosphoinositides in control of fission and fusion

Binding dynamin much tighter than PI4P, PI(4,5)P_2_ was implicitly assumed essential for dynamin-mediated fission (*48*–*52*), whereas PI4P was considered merely its precursor. Our results suggest modifying the dynamin-mediated fission model by including (i) PI4P as the essential phospholipid and (ii) rapid calcium/synaptojanin-induced PI(4,5)P_2_-to-PI4P transition for initiating fission. This model does not contradict PI(4,5)P_2_’s role in endocytic pit formation preceding fission (*53*, *54*). It is consistent with, yet may mechanistically explain, early observations implicating PI(4,5)P_2_ turnover in endocytosis and/or fission. These early observations include inhibition of synaptic vesicle endocytosis, vesicle recycling and/or clathrin-mediated endocytosis by synaptojanin 1 or 2 knockout or knockdown (*55*–*58*), and increased membrane fragmentation after recruiting synaptojanin 1 5-phosphatase domain to endophilin-induced tubules (*59*). The present work advances over these early studies by revealing rapid calcium-induced, synaptojanin-mediated PI(4,5)P_2_-to-PI4P transition and its resulting PI4P increase with concurrent PI(4,5)P_2_ decrease in initiating dynamin-mediated pre-Ω/fs-Ω pore closure, which underlies diverse endocytic modes in live cells. Our model may thus apply to various cell types used in previous synaptojanin studies.

Binding SNARE proteins and synaptotagmin, PI(4,5)P_2_ is essential for vesicle priming and fusion (*23*, *60*–*62*) [but see (*63*)]. Our results suggest modifying this phosphoinositide-relevant fusion model by including a crucial role of the calcium-induced, synaptojanin-mediated PI(4,5)P_2_-to-PI4P transition and its resulting PI4P-to-PI(4,5)P_2_ ratio increase in promoting fusion pore constriction/closure that underlies kiss-and-run fusion.

In summary, our findings provide insights into the fundamental initiation mechanisms of membrane fission, kiss-and-run fusion, and endocytosis, as well as the pathogenesis of disorders associated with impaired phosphoinositide metabolism. The concept of initiating fission and endocytosis through a single, rapid phosphoinositide metabolic transition, as revealed here, provides valuable insight into how complex molecular machinery is triggered in general.

## MATERIALS AND METHODS

### Chromaffin cell culture

Bovine adrenal chromaffin cells containing dense-core vesicles are widely used for exo-endocytosis studies (*4*, *64*). To prepare primary bovine adrenal chromaffin cell culture (*9*, *16*), we purchased fresh bovine adrenal glands from a local abattoir (J. W. Treuth & Sons Inc., 328 Oella Ave, Catonsville, MD 21228; https://jwtreuth.com), which collected the adrenal glands immediately after the animal’s death. The use of bovine adrenal glands for the culture of adrenal chromaffin cells did not require an animal use protocol. Fresh adult (21 to 27 months old) bovine adrenal glands were immersed in prechilled Locke’s buffer on ice for transportation to the lab. This solution contained: NaCl, 145 mM; KCl, 5.4 mM; Na_2_HPO4, 2.2 mM; NaH_2_PO4, 0.9 mM; glucose, 5.6 mM; and HEPES, 10 mM (pH 7.3, adjusted with NaOH). Upon arrival at the lab, glands were perfused with Locke’s buffer, then infused with Locke’s buffer containing collagenase P (1.5 mg/ml, Roche), trypsin inhibitor (0.325 mg/ml, Sigma-Aldrich) and bovine serum albumin (5 mg/ml, Sigma-Aldrich), and incubated at 37°C for 20 min. The digested medulla was minced in Locke’s buffer and filtered through a 100-μm nylon mesh. The filtrate was centrifuged (48*g*, 5 min), resuspended in Locke’s buffer and recentrifuged until the supernatant was clear. The final cell pellet was resuspended in prewarmed Dulbecco’s Modified Eagle medium (Gibco) supplemented with 10% fetal bovine serum (Gibco) and plated onto poly-l-lysine (0.005% w/v, Sigma-Aldrich) and laminin (4 μg/ml, Sigma-Aldrich) coated glass coverslips.

### Electroporation and plating for chromaffin cell culture

Cells were transfected by electroporation using Basic Primary Neurons Nucleofector Kit (Lonza), according to the manufacturer’s protocol and plated onto glass coverslips with mouse Laminin coating over poly-D-lysine layer (Neuvitro). The cells were incubated at 37°C with 9% CO_2_ and used within 5 days.

### Plasmids, siRNA and fluorescent dyes for chromaffin cells

When A655 (Sigma-Aldrich) was included in the bath solution, the dye concentration was 30 μM. PH-EGFP (phospholipase C delta PH domain attached with EGFP) was obtained from T. Balla. PH-mNeonGreen (PH_G_), PH-mTFP1, PH-mCherry (PH_mCh_), and PH-SNAP constructs were generated by replacing the EGFP tag of PH-EGFP with mNeonGreen (Allele Biotechnology) (*65*), mTFP1 (Addgene), mCherry (Addgene), and the SNAP tag (Addgene), respectively. For FFN511 or FFN206 (Abcam) imaging, cells were bathed with FFN511 or FFN206 (5 to 10 μM) for 20 min and images were performed after washing out FFN511 or FFN206 in the bath solution. Dynamin 2–mTFP1 (or dynamin 1–mTFP1) and dynamin 2–mNeonGreen constructs were created by replacing the EGFP tag of dynamin 2–EGFP (or dynamin 1–EGFP, Addgene) with mTFP1 or mNeoGreen, respectively. P4M-EGFP was obtained from Addgene (no. 51472); P4M-mTFP1 and P4M-SNAP were created by replacing EGFP tag of P4M-EGFP with mTFP1 or the SNAP tag, respectively. OSBP-EGFP was obtained from T. Balla.

The plasmids PJ-Sac [FKBP (FK506 binding protein 12) attached with *S. cerevisiae* sac1 phosphatase, inactivated mutation (Asp^1263^Ala) in the INPP5E domain, and mCherry (Addgene, no. 38000)], PJ-dead [FKBP attached with inactivated *S. cerevisiae* sac1 phosphatase, inactivated mutation (Asp^1263^Ala) in the INPP5E domain, and mCherry (Addgene, no. 38002)], and Lyn_11_-FRB (Lyn_11_ FKBP-rapamycin binding domain from mTOR; Addgene, no. 38004) were obtained from Addgene. The plasmid mCherry-CRY2-5′Ptase_(OCRL)_-T2A-CIBNcaax (also referred to as CIBN/CRY2-OCRL in the present work for simplicity) contains CIBN-CAAX (amino acids 1 to 170 of *A. thaliana* CIB1 attached with CAAX) and cryptochrome 2 (CRY2) attached with mCherry (for recognition) and an inositol polyphosphate-5-phosphatase domain of OCRL. This plasmid obtained from X. Lou (*23*). Another plasmid, mCherry-CRY2-5′Ptase_OCRL_(D523G)-T2A-CIBNcaax (also referred to as CIBN/CRY2-OCRL_mut_), which was also obtained from X. Lou (*23*), is similar to mCherry-CRY2-5′Ptase_(OCRL)_-T2A-CIBNcaax except that OCRL is replaced with D523G OCRL. This OCRL mutant could not dephosphorylate PI(4,5)P_2_ into PI4P but may act as a dominant negative competing with and thus inhibiting endogenous phosphatases that mediate PI(4,5)P_2_-to-PI4P conversion (*23*).

For knockdown of endogenous Synj1 in bovine chromaffin cells, one siRNA duplex for bovine Synj1 (5′-CAAUAACAAUUACUUUAAAdTdT-3′) labeled with cyanine 5 and scrambled control siRNA (SIC003) were purchased from Sigma-Aldrich. Synj1-mCherry construct was created by replacing EGFP tag of Synj1-EGFP (Addgene, no. 22293) with mCherry. Synj1 R839C mutant was generated from Synj1-mCherry plasmid.

### Assessment of PH domain overexpression effects on exocytosis

It was reported that overexpression of the phospholipase C delta PH domain inhibits exocytosis induced by a train of 200 ms depolarizations in chromaffin cells (*60*), but does not inhibit glucose-induced insulin secretion in MIN6 cells (*66*). To determine whether PH_G_ overexpression affects exocytosis in our recording system, we compared capacitance jumps and the peak of calcium currents between cells with and without PH_G_ expression. No significant differences were observed (fig. S16), indicating that PH domain expression does not impair exocytosis induced by 1-s depolarization under our experimental conditions. It is likely that different stimulation protocols might explain differential impacts of PH_G_ overexpression on exocytosis.

### Western blot

Total protein was extracted from cultured chromaffin cells using RIPA buffer containing protease inhibitor cocktail (Millipore Sigma). Equal amounts of protein, determined by BCA protein assay (Invitrogen) were loaded onto 4 to 12% bis-tris gel (Invitrogen). Proteins were transferred onto polyvinylidene difluoride membrane and immunoblotted with indicated primary antibodies at 4°C overnight. Membranes were incubated with horseradish peroxidase labeled secondary antibodies and visualized using Bio-Rad ChemiDoc Imaging System. Primary antibodies including anti-Synaptojanin 1 (1:500, Cell Signaling Technology) and β-actin (1:3000; Abcam).

### Chemical-genetic and optogenetic approaches to reduce PI4P and PI(4,5)P_2_

We reduced PI4P level at the PM with a chemical-genetic approach using rapamycin-inducible dimerization between PM-anchored Lyn_11_-FRB and FKBP-linked PJ-Sac that dephosphorylates PI4P (*29*). FKBP-linked PJ-dead, in which the mutant sac1 could not dephosphorylate PI4P, served as the control (*29*). Rapamycin application to cells transfected with Lyn_11_-FRB and FKBP-linked PJ-Sac (referred as PJ-Sac–transfected cells for simplicity), but not cells transfected with Lyn_11_-FRB and FKBP-linked PJ-dead (referred as PJ-dead–transfected cells for simplicity), reduced PI4P at PM (Fig. 4 and figs. S10 and S11) (*29*).

We optogenetically reduced PI(4,5)P_2_ using light-induced dimerization between cytosolic CRY2 and PM-localized CIBN-CAAX, where CRY2 was attached with mCherry (for recognition) and OCRL to dephosphorylate PI(4,5)P_2_ (*23*). Blue-light exposure to CIBN/CRY2-OCRL–transfected cells reduced PI(4,5)P_2_ at PM (Fig. 4 and fig. S12) (*23*).

### Electrophysiology and solutions for chromaffin cells

At room temperature (20° to 22°C), whole-cell voltage clamp and capacitance recordings were performed with an EPC-10 amplifier together with the software lock-in amplifier (PULSE 8.74, HEKA, Lambrecht, Germany) (*9*, *67*). The holding potential was −80 mV. For capacitance measurements, the frequency of the sinusoidal stimulus was 1000 to 1500 Hz with a peak-to-peak voltage ≤50 mV. The bath solution contained 125 mM NaCl, 10 mM glucose, 10 mM HEPES, 5 mM CaCl_2_, 1 mM MgCl_2_, 4.5 mM KCl, 0.001 mM tetrodotoxin, and 20 mM tetraethylammonium (pH 7.3) adjusted with NaOH. The pipette (2 to 4 megohms) solution contained 130 mM Cs-glutamate, 0.5 mM Cs-EGTA, 12 mM NaCl, 30 mM HEPES, 1 mM MgCl_2_, 2 mM adenosine 5′-triphosphate, and 0.5 mM guanosine 5′-triphosphate (pH 7.2) adjusted with CsOH. These solutions pharmacologically isolated calcium currents.

PAO (20 μM, 10 min, Merck) was applied to the bath solution in some experiments; rapamycin (0.5 μM, 5 to 20 min, Merck) was applied to the bath solution in some experiments; GSK-A1 (100 nM, 10 min, MCE) was applied to the bath solution in some experiments

For stimulation, we used a 1-s depolarization from the holding potential of −80 to +10 mV (depol_1s_). We used this stimulus because it induces robust exo-endocytosis as reflected in capacitance recordings (Fig. 1A) (*9*, *68*, *69*). Since prolonged whole-cell recording slows down endocytosis (*70*), we limited to 1 depol_1s_ per cell. To quantify Ca^2+^ (or Sr^2+^) entry, peak Ca^2+^ (or Sr^2+^) current amplitude was measured as the maximum inward current observed during the 1-s depolarization.

### Confocal imaging of chromaffin cells

Imaging of PH_G_ and A655 was performed with an inverted confocal microscope (TCS SP5II, Leica, Germany, 100× oil objective, numerical aperture of 1.4). PH_G_ was excited by a tunable white-light laser at 515 nm (laser power set at ~1 to 4 mW); A655 was excited by an HeNe laser at 633 nm (laser power set at ~12 to 15 mW); their fluorescence was collected at 520 to 600 and 650 to 800 nm, respectively. Confocal imaging area was ~70 to 160 μm^2^ at the *XY* plane with a fixed *Z*-axis focal plane ~100 to 200 nm above the cell-bottom membrane (*XY*/*Z*_fix_ scanning). Images were collected every 40 to 80 ms at 40 to 60 nm per pixel. For imaging of PH_G_, A655, and FFN511, all settings were the same as described above except that FFN511 was excited by an argon laser at 458 nm (laser power set at ~2 to 4 mW), and its fluorescence was collected at 465 to 510 nm.

When PI(4,5)P_2_ was optogenetically reduced, Close_pre-Ω_ and Close_fs-Ω_ were detected with imaging of P4M-EGFP, A655, and FFN511 (Fig. 4F and S13). In these experiments, we replaced PH_G_ with P4M-EGFP because PI(4,5)P_2_ reduction significantly reduced PM PH_G_ fluorescence (Fig. 4D). P4M-EGFP was excited by a tunable white-light laser at 496 nm (laser power set at ~1 to 4 mW); FFN511 was excited by an argon laser at 458 nm (laser power set at ~2 to 4 mW); A655 was excited by an HeNe laser at 633 nm (laser power set at ~12 to 15 mW); their fluorescence was collected at 505 to 580, 465 to 490, and 650 to 800 nm, respectively.

### Detection of preformed Ω-profile pore closure, fusion pore closure, and various fusion modes

At rest, preformed PH_G_ spots (or rings) overlapped with A655 spots (prespots) were observed at the cell bottom (Fig. 2, A to D), which reflected mostly (88 ± 3%, 13 cells) Ω-shape profiles, but sometimes Λ-shape, as verified with *XZ*-plane STED microscopy [Fig. 2A, see also (*10*)]. Close_pre-Ω_ was reflected as A655 fluorescence (F_655_, strongly excited) dimming, while PH_G_ fluorescence (F_PH_, weakly excited) sustained or dimmed with a delay (Fig. 2, B to D), due to pore closure that prevented bleached A655 (by strong excitation) from exchanging with bath fluorescent A655 [see (*9*, *10*, *15*, *16*) for detail]. The detected pore closure is impermeable to H^+^ and OH^−^, mediated by dynamin, and directly observed with STED imaging (fig. S7) (*9*, *10*, *15*, *16*). It reflects mostly pre-Ω pore closure, as preformed Λ closure was negligible (*10*).

Vesicle fusion was identified as the sudden appearance of PH_G_ (or P4M-EGFP) spot together with the sudden appearance of an A655 spot, due to PH_G_ (or P4M-EGFP) and A655 diffusion from the PM and the bath into the fusion-generated Ω-profile (fs-Ω, Fig. 3, A to C) (*9*, *10*, *16*). Fs-Ω detected with this method was verified with direct observation from STED imaging (e.g., fig. S8) and with concurrent imaging of fluorescent false neurotransmitter FFN511 (preloaded in vesicles) at the same spot (*10*, *15*), which showed FFN511 fluorescence decrease as F_PH_ and F_655_ increased (Fig. 3, A to C). In this study, FFN511 imaging was combined with imaging of PH_G_ (or P4M-EGFP) and A655 in some experiments for measurements of FFN511 release rate.

Fs-Ω may close its pore at ~0.05 to 30 s later (Close_fs-Ω_, also called close fusion or kiss-and-run, Fig. 3A), maintain an open pore (stay fusion, Fig. 3B), or shrink to merge with the PM (shrink fusion, Fig. 3C) (*15*, *26*). Close_fs-Ω_ was detected as A655 fluorescence (F_655_, strongly excited) dimming due to pore closure that prevented bath fluorescent A655 from exchanging with bleached A655, while F_PH_ (weakly excited) sustained or decayed with a delay that reflected PI(4,5)P_2_ conversion into PI4P and/or vesicle pinch off (Fig. 3A); stay fusion was detected as sustained F_655_ and F_PH_ (Fig. 3B); in shrink fusion, A655 and PH_G_ spots are shrinking with parallel decreases of F_655_ and F_PH_ (Fig. 3C) (*15*, *16*, *26*).

Pore closure (Close_fs-Ω_ and Close_pre-Ω_) detected with spot F_655_ bleaching by strong excitation is not due to a narrow pore smaller than A655 molecule size, because after spot dimming, bath application of an acid solution cannot quench the pH-sensitive VAMP2-EGFP or VAMP2-pHluorin overexpressed at the same spot, indicating that the spot is impermeable to H^+^ or OH^−^, the smallest molecules, and thus is closed (*9*, *10*, *16*). Furthermore, pore closure detected with this method was blocked by dynamin inhibitor dynasore, dynamin dominant-negative mutant dynamin 1-K44A, or dynamin knockdown, suggesting that Close_pre-Ω_ and Close_fs-Ω_ are mediated by dynamin (*9*, *10*, *16*).

When PH_G_ was replaced with P4M-EGFP (Fig. 4F), detection of Close_pre-Ω_, Close_fs-Ω_ (close fusion), stay fusion, and shrink fusion was similar to that with PH_G_ imaging (fig. S13). Prespots were detected as preformed P4M-EGFP spots overlapping with A655 spots; Close_pre-Ω_ was detected as A655 fluorescence (F_655_, strongly excited) dimming, while P4M-EGFP fluorescence (F_P4M_) sustained or dimmed with a delay (fig. S13A). Fusion was identified as the sudden appearance of P4M-EGFP spot together with the sudden appearance of an A655 spot, which was further confirmed with concurrent imaging of FFN511 release from the same spot (fig. S13B). Close_fs-Ω_ was detected as A655 fluorescence (F_655_, strongly excited) dimming due to pore closure that prevented bath fluorescent A655 from exchanging with bleached A655, while F_P4M_ (weakly excited) sustained or decayed with a delay; stay fusion was detected as sustained F_655_ and F_P4M_ (fig. S13B).

### Assessment of PI4P effects on vesicle docking and fusion

We determined whether PI4P affects exocytosis by comparing the depol_1s_-induced capacitance jumps in control, in the presence of GSK-A1 or PAO that substantially reduced PI4P, and in synaptojanin knockdown or mutation that substantially inhibits depol_1s_-induced PI4P increase. We found no significant differences in all these conditions (figs. S17 and S18), suggesting that PI4P does not affect the exocytosis amount.

We also quantified FFN511-labeled vesicles at the PM, as detected with confocal microscopy at the *XY* plane at the cell-bottom membrane (fig. S18A). We found no significant differences in the vesicle numbers among control cells, cells with Si-Synj1, or with Synj1_mut_ overexpression (fig. S8B). Thus, synaptojanin does not affect vesicle recruitment to dock at the PM (fig. S18, A and B) or fusion (fig. S18, C and D).

### MINFLUX nanoscopy

#### Cell preparation and SNAP labeling

Chromaffin cells transfected with PH-SNAP or P4M-SNAP were fixed with paraformaldehyde (PFA; 2.4%) and sucrose (2.4%) solution for 30 min. Excess PFA was quenched with 50 mM NH_4_Cl solution for 10 min, followed by a 30-min incubation with Image-iT Signal Enhancer solution at room temperature. Subsequently, cells were incubated at room temperature for 50 min with SNAP substrate dye solution containing 1 μM SNAP-Surface Alexa Fluor 647 (NEB, S9136S), 0.5% bovine serum albumin, and 1 mM dithiothreitol (DTT), resulting in the attachment of SNAP-surface Alexa Fluor 647 to PH-SNAP (PH-SNAP-A647) or P4M-SNAP (P4M-SNAP-A647), which were used for MINFLUX imaging. MINFLUX imaging resolves individual PH-SNAP or P4M-SNAP molecules that reflect PI(4,5)P_2_ or PI4P at the pore region in the resting condition (Fig. 5, C and D).

Next, an undiluted dispersion of gold beads (EM.GC150/4, BBI Solutions) was incubated for 10 min and rinsed of several times with phosphate-buffered saline to remove unbound gold beads. The coverslips containing chromaffin cells were mounted on a depression slide with the MINFLUX imaging buffer containing 50 mM tris/HCl, 10 mM NaCl, 10% (w/v) glucose, catalase (64 μg/ml), glucose oxidase (0.4 mg/ml), and 15 mM MEA at pH 8.0. The coverslips were sealed with Elite double 22 dental epoxy (Zhermack).

#### MINFLUX data acquisition

MINFLUX imaging was performed on a commercial 3D MINFLUX microscope that was driven by the Imspector software with MINFLUX drivers (Abberior Instruments) (*71*). Typically, fields of view containing numerous gold beads were selected and fixed in a three-dimensional configuration to stabilize the sample during experimentation. This stabilization was achieved by using the near-infrared scattering from gold beads and active feedback correction via the piezo stage. It was ensured that the SD of the sample position relative to the stabilization set point was less than 2 nm in all directions during measurements. Fields of view were chosen close to the coverslip surface at the bottom of the cell expressing both fusion proteins. Before starting the MINFLUX data acquisition, iterative confocal scans were performed using a 640-nm excitation laser and power between 8 to 15% (maximum power at periscope, 1.94 mW) to drive the fluorophores to the dark state. The sample was imaged with the standard MINFLUX imaging sequence provided by the manufacturer using 3 to 5% fixed laser power. During the MINFLUX measurement, the 405-nm activation laser power was ramped up slowly from 0 to 50% over several hours (maximum power at periscope, 27 μW). Samples were typically imaged for 8 to 12 hours.

#### MINFLUX data analysis

The raw final valid molecule position estimates were exported directly from the MINFLUX Imspector interface as a .mat file. Matlab code was used to identify and segregate clusters of localizations based on their trace ID. Traces originating from the same fluorophore emission burst were filtered out if they contained less than three localizations per trace (*72*, *73*). MINFLUX localization images were then generated.

### STED imaging of chromaffin cells

STED images were acquired with Leica TCS SP8 STED 3× microscope that is equipped with a 100 × 1.4 numerical aperture HC PL APO CS2 oil immersion objective and operated with the LAS-X imaging software. Excitation was with a tunable white-light laser and emission was detected with hybrid (HyD) detectors. PH_G_ and A532 were sequentially excited at 485 and 540 nm, respectively, with the 592-nm STED depletion beam, and their fluorescence collected at 490 to 530 and 545 to 587 nm, respectively.

For three-color STED imaging with 592-nm STED depletion laser, mTFP1, mNeonGreen, and A532 were excited at 442 (power, 1 to 3 mW), 507 (power, 1 to 5 mW), and 545 nm (power, 4 to 6 mW), respectively, and their fluorescence was collected at 447 to 502, 512 to 540, and 550 to 587 nm, respectively. In some experiments we performed three-color STED imaging on FFN206, P4M-mTFP1, dynamin 2-mNeonGreen by exciting them at 405 (power, 10 to 15 mW), 470 (power, 1 to 5 mW), and 505 nm (power, 1 to 5 mW), respectively, and collecting fluorescence at 410 to 465, 475 to 500, and 515 to 570 nm, respectively.

For two-color STED imaging of mTFP1 and mNeonGreen with the 592-nm STED depletion beam, mTFP1 and mNeonGreen were sequentially excited at 442 (power, 1 to 3 mW) and 507 nm (power, 1 to 5 mW), respectively, and their fluorescence collected at 447 to 505 and 516 to 587 nm, respectively. STED *XZ*-plane imaging was performed with 3D depletion pattern [z(3D)-STED]. The depletion laser power distribution of depletion 3D doughnut was 60% or higher in *Z* direction and 40% or lower in *XY* direction. The depletion laser power was 100 to 500 mW for imaging of mNeonGreen and mTFP1 but 100 to 200 mW for imaging of A532.

STED images were acquired at the cell bottom at the *XZ* plane with the *Y* axis fixed at about the center of the cell. Images were acquired every 26 to 200 ms at 15 nm per pixel in an *XZ* area of 15 to 20 μm × 0.7 to 2.5 μm. The imaging duration was limited to 10 to 20 s before and 60 s after depol_1s_. We limited to 60 s, because whole-cell endocytosis after depol_1s_, measured with capacitance recordings, usually takes place within 60 s (e.g., Fig. 1A) (*9*, *15*, *16*). Each cell was subjected to only 1 depol_1s_ to avoid endocytosis rundown (*70*).

The STED resolution for imaging PH_G_ in our conditions was ~60 nm on the microscopic *X* and *Y* axes (parallel to cell-bottom membrane or coverslip), and ~150 to 200 nm on the microscopic *Z* axis. STED images were deconvolved using Huygens software (Scientific Volume Imaging) and analyzed with ImageJ and LAS X (Leica).

During STED *XZ*/*Y*_fix_ imaging, A532 was excited at a high laser power so that fluorescent A532 can be bleached with a time constant of 1.5 to 3.5 s. Pore closure was identified as the gradual dimming of the A532 spot fluorescence to baseline during *XZ*/-plane PH_G_/A532 imaging. A532 fluorescence dimming is due to pore closure that prevents bleached A532 (by strong excitation) from exchange with a large reservoir of fluorescent A532 (very small molecule, ~1 nm) in the bath.

### EM on liposomes incubated with dynamin and PI4P or PI(4,5)P_2_

#### Liposome preparation

Lipids with the composition of 68% 1,2-dioleoyl-*sn*-glycero-3-phosphocholine (DOPC), 30% DOPS, 2% l-α-phosphatidylinositol-4,5-bisphosphate [PI(4,5)P_2_] or 2% l-α-phosphatidylinositol-4-phosphate (PI4P) (Avanti Polar Lipids) were dried and resuspended in 250 μl of HCB150 (50 mM HEPES, 150 mM KCl, 2 mM EGTA, 1 mM MgCl_2_, and 1 mM DTT, pH 7.5). Unilamellar liposomes were obtained by extruding the mixture 21 times through a 0.8-μm pore size polycarbonate membrane (Avanti) (*74*).

#### Expression and purification of dynamin 1 ΔPRD construct

Recombinant dynamin 1 ΔPRD was cloned into a pET47b vector with an N-terminal 6xHis tag upstream of an HRV 3C proteolytic cleavage site and expressed in *Escherichia coli* BL21. Culture was grown to optical density at 600 nm = 0.6, cold shocked on ice for 30 min, and protein expression induced using 0.1 mM isopropyl-β-d-thiogalactopyranoside. Cells were harvested after 20 hours of growth at 18°C with shaking at 180 RPM and cell pellets stored at −80°C. Pellets were thawed in 50 ml of lysis buffer [50 mM HEPES (pH 8.0), 150 mM KCl, 10 mM imidazole, and 5 mM β-mercaptoethanol] with protease inhibitors (Roche). Resuspended cells were incubated at 4°C for 30 min and lysed using sonication (total time of 10 min with 10-s pulse on and 15-s pulse off). Lysate was centrifuged for 1 hour at high-speed centrifugation (20,000*g*, 15 min) and the supernatant was purified by Ni–nitrilotriacetic acid (NTA) affinity chromatography. To remove the 6xHis tag, dynamin protein fractions were combined, treated with 10xHis-tagged HRV 3C protease, and dialyzed overnight at 4°C into modified HCB150 buffer [20 mM HEPES (pH 7.3), 150 mM KCl, 2 mM EGTA, 1 mM MgCl_2_, 5 mM BME, and 5.7 mM imidazole]. Uncleaved His tag–dynamin was removed by another round of Ni-NTA chromatography. Fractions containing cleaved dynamin were pooled and concentrated to 500 μl using an Amicon centrifugal filter unit. The sample was further purified by an S650 size exclusion column (Bio-Rad) with HCB150 buffer. Fractions containing dynamin at >95% purity by SDS–polyacrylamide gel electrophoresis and Coomassie staining were pooled, flash frozen in liquid nitrogen, and stored at −80°C.

#### Dynamin tubes preparation

Dynamin decorated tubes with different lipids were generated by incubating liposomes with protein (0.8 mg/ml, in HCB150) for 1 hour. The tubes were then analyzed for diameter measurements by negative stain and cryo-EM (*74*).

#### Negative stain

Dynamin decorated tubes were loaded onto carbon film 300 mesh, copper grids (CF300-CU), and incubated for 1 min, followed by staining with 1% uranyl acetate. Grids were visualized on an FEI Technai 12 microscope at 18,000 × nominal magnification, as part of the National Institute of Diabetes and Digestive and Kidney Diseases (NIDDK) core facility. The outer diameter measurement of samples was calculated in Fiji (*75*).

#### Cryo-EM

Each sample (3.5 μl) was applied to the plasma-cleaned Quantifoil R 1.2/1.3, Cu 200 grids (Electron Microscopy Sciences, catalog no. Q2100CR1.3), preblotted (30 s), and blotted with filter paper (Whatman 1) for 2 s followed by plunging into liquid ethane using a Leica EM GP (Leica Microsystems). The samples were stored in liquid nitrogen until data acquisition. Data were collected on a 200 kV Thermo Scientific Glacios transmission electron microscope at 36,000× nominal magnification (calculated pixel size is 0.56 Å), with a target defocus range of −1.8 to −3.0 μm using a K3 direct detection camera (Gatan Inc.). The diameter measurement of samples was calculated in Fiji (*75*).

### Data selection

For every cell recorded with a pipette under the whole-cell configuration, the data within the first 2 min at the whole-cell configuration were used, which avoided rundown of endocytosis (gradual disappearance of endocytosis) as previously reported under the whole-cell configuration for a long time (*9*, *70*).

Confocal and STED images were analyzed with ImageJ and LAS X (Leica). The fluorescence intensity from an area covering the fluorescence spot was measured at every image frame. The full width half maximum (W_H_) was measured from intensity profiles of one to four lines across the spot center.

### Statistical tests

Data were expressed as mean ± SEM. Replicates are indicated in results and figure legends. *N* represents the number of cells, fusion events, or experiments as indicated in results and figure legends. The statistical test used is *t* test or analysis of variance (ANOVA). Although the statistics were performed on the basis of the number of cells, fusion events, and Close_pre-Ω_, each group of data were replicated from at least four primary chromaffin cell cultures. Each culture was from at least three to four glands from two bovines.

## Supplementary Materials

### This PDF file includes:

Figs. S1 to S18 References
