# Supplementary material for: A phosphoinositide switch from PI(4,5)P2 to PI4P triggers endocytosis by inducing dynamin-mediated fission in secretory cells
Source: Sci Adv. 2025 Oct 15;11(42):eady8065. doi: 10.1126/sciadv.ady8065 (PMC12526769; doi:10.1126/sciadv.ady8065)
Supplement: Supplementary file 1 — Figs. S1 to S18 References [file sciadv.ady8065_sm.pdf]

Science Advances
AAAS

Supplementary Materials for

A phosphoinositide switch from PI(4,5)P_2 to PI4P triggers endocytosis by inducing dynamin-mediated fission in secretory cells

Xiaoli Guo *et al.*

Corresponding author: Jenny E. Hinshaw, jennyh@niddk.nih.gov; Ling-Gang Wu, wul@ninds.nih.gov

Sci. Adv. **11**, eady8065 (2025)
DOI: 10.1126/sciadv.ady8065

This PDF file includes:

Figs. S1 to S18
References

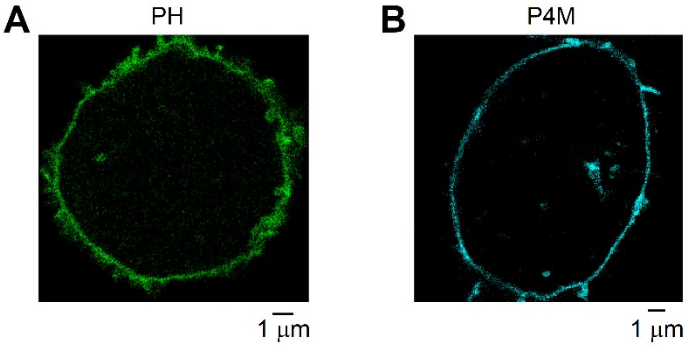

Fig. S1. PI(4,5)P_2 and PI4P detected along the cell outline when the focal plane was aligned with the cell center.

(**A-B**) Confocal XY-plane image showing PH_G-labeled PI(4,5)P_2 (A) or P4M-EGFP-labeled PI4P (B) localized at the plasma membrane, with the focal plane aligned to the cell center.

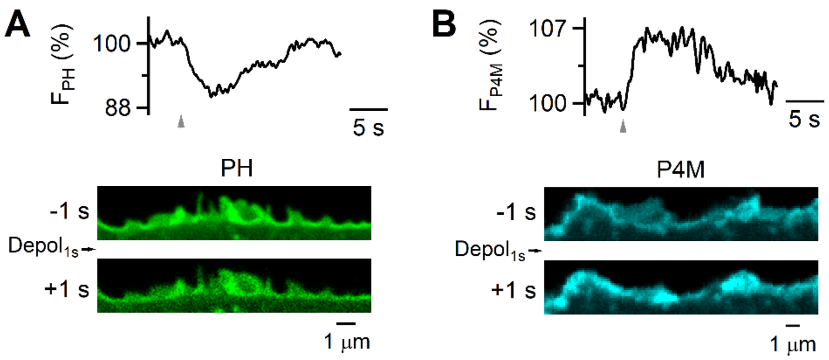

Fig. S2. Depolarization induces F_{PH}-decrease and F_{P4M}-increase as observed with confocal XY-plane imaging at the cell outline.

(**A-B**) Changes in F_{PH} (A) and F_{P4M} (B) induced by $depol_{1s}$ (gray triangle), with representative confocal XY-plane images sampled before (−1 s) and after (+1 s) $depol_{1s}$ from a zoomed-in region of the cell. A and B are from different cells and are different from cells shown in Figure S1.

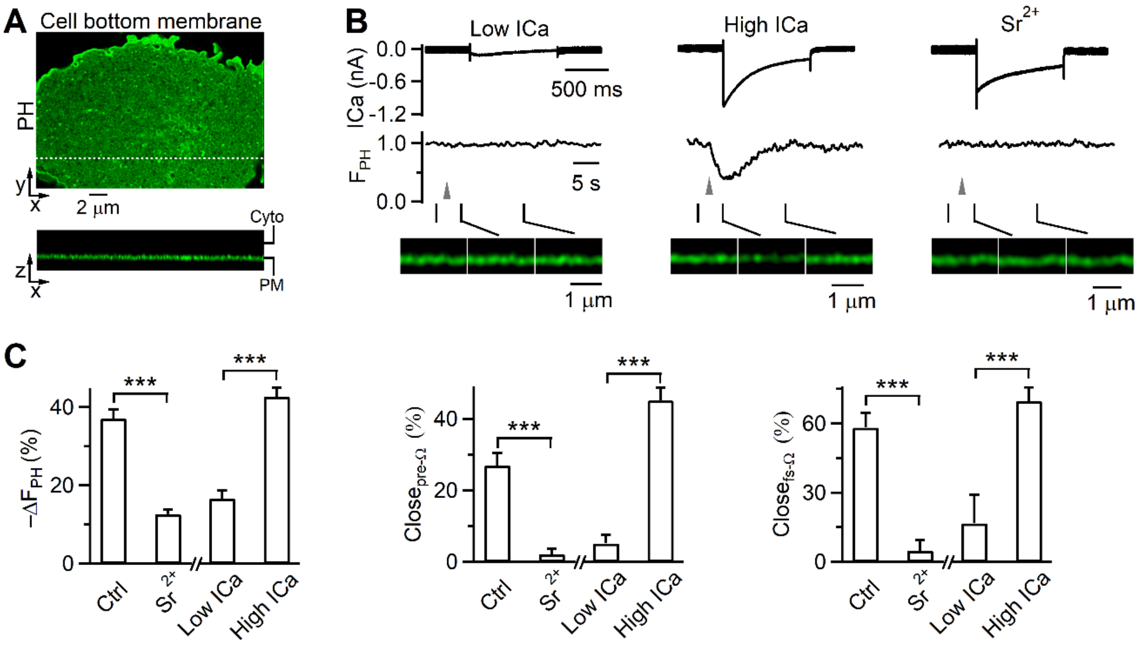

Fig. S3. Depolarization-induced calcium influx triggers F_{PH}-decrease as observed with STED XZ-plane imaging.

(**A**) Upper: XY-plane confocal image showing PH_G-labeled PI(4,5)P_2 at the plasma membrane at the cell bottom above the coverslip.
Lower: XZ-plane image of the white dotted line in the upper panel showing the thin cell-bottom plasma membrane (PM) containing PH_G-labeled PI(4,5)P_2. Cyto: cytosol above the cell-bottom PM.

(**B**) ICa (upper) and F_{PH} (middle) induced by $depol_{1s}$ (gray triangle), and sampled STED XZ-plane images of PH_G at times indicated by lines in a cell with a low ICa (left), a cell with a high ICa (middle) and a cell with the extracellular calcium being replaced with strontium (Sr^{2+}, 5 mM, right).

(**C**) Percentage of F_{PH}-decrease ($-\Delta F_{PH}$), $Close_{pre\text{-}\Omega}$ and $Close_{fs\text{-}\Omega}$ in control cells (Ctrl, n = 55), cells in which extracellular calcium was replaced with strontium (Sr^{2+}, 5 mM, n = 40), the low-ICa subgroup within the control group (ICa < 400 pA, n = 12), and the high-ICa subgroup within the control group (ICa > 400 pA, n = 43). Data expressed as mean + s.e.m. ***: $p < 0.001$, t test.

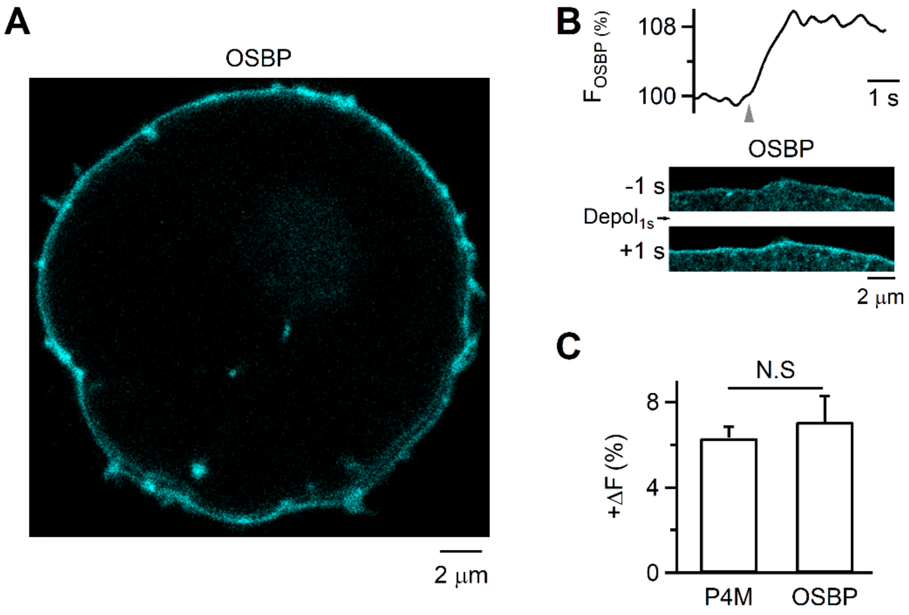

Fig. S4. OSBP-EGFP exhibited spatial and temporal dynamics similar to those of P4M-EGFP following 1-s depolarization.

(**A**) Confocal XY-plane image showing OSBP-EGFP-labeled PI4P localized at the plasma membrane, with the focal plane aligned to the cell center.

(**B**) Changes in OSBP-EGFP fluorescence (F_{OSBP}, upper) induced by depol$_{1s}$ (gray triangle), with sampled confocal XY-plane images (lower) before (−1 s) and after (+1 s) depol$_{1s}$ from a zoomed-in region of the cell. A and B are from different cells.

(**C**) The depol$_{1s}$-induced fluorescence increase (+ΔF) of P4M-EGFP (36 cells) and OSBP-EGFP (10 cells); data normalized to the baseline before depol$_{1s}$ and presented as the percentage (mean + s.e.m.). N.S: not significant, $p > 0.05$ (t-test).

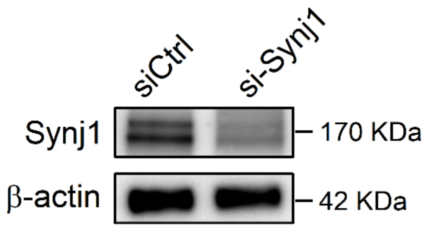

Fig. S5. Representative immunoblotting showing knockdown of synaptojanin 1 in primary chromaffin cell cultures.

Chromaffin cells transfected with scrambled (siCtrl) or Si-Synj1 were tested for the expression of synaptojanin 1 (Synj1) by western blotting. β-actin was used as the loading control. The molecular weight of protein markers is indicated. Two Synj1 bands in the western blot are consistent with the existence of two major Synj1 isoforms (*76*).

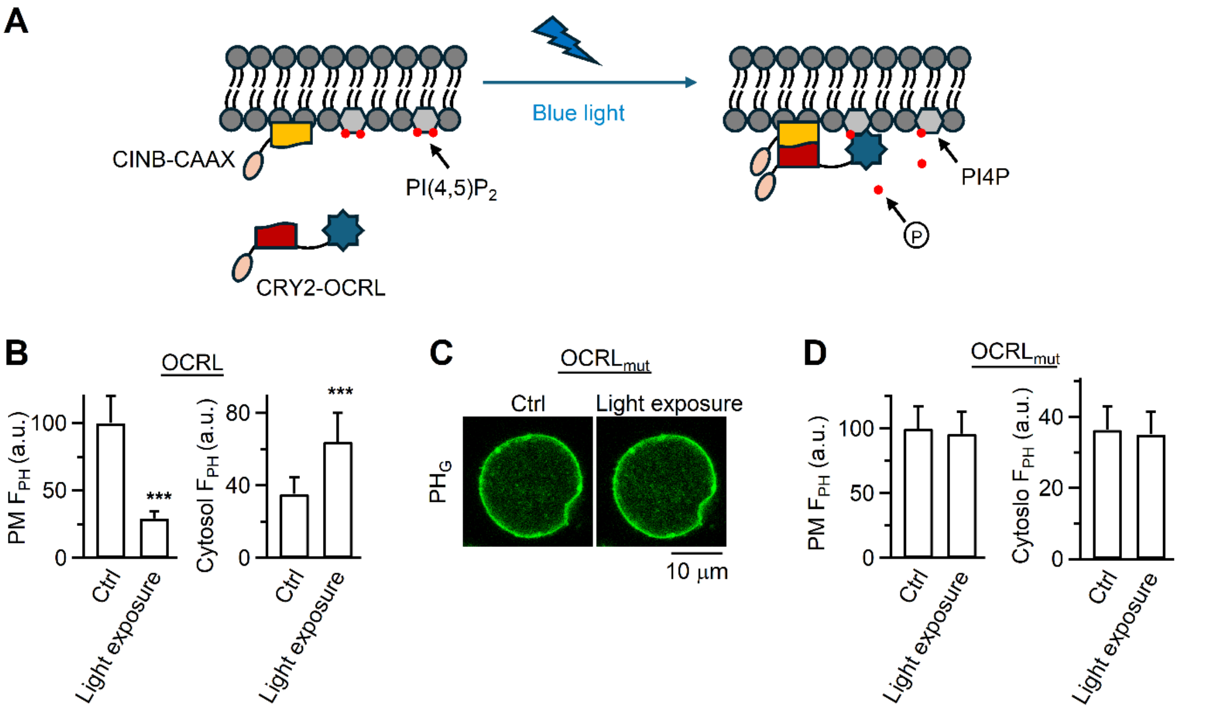

Fig. S6. Mutant OCRL does not affect PI(4,5)P$_2$ level.

(**A**) Schematic drawing showing CRY2-OCRL and the membrane-targeted CIBN-CAAX before and after blue light exposure – after light exposure, OCRL is recruited to the plasma membrane to convert PI(4,5)P$_2$ into PI4P.

(**B**) Quantification of F_{PH} at PM (plasma membrane) and in the cytosol before (Ctrl) and after a blue light exposure in cells transfected with mCherry-attached CIBN-CAAX/CRY2-OCRL (mean + s.e.m., 10 cells). a.u.: artificial unit. ***: $p < 0.001$, paired t test. The increase in cytosolic F_{PH} reflects PH-GFP dissociation from the plasma membrane due to light-induced depletion of PI(4,5)P$_2$. PM F_{PH} plot is the same as the bar graph in Fig. 4D in the main text.

(**C**) Sampled PH_G (labeling PI(4,5)P$_2$) before (Ctrl) and after blue light exposure to a cell transfected with CIBN-CAAX/CRY2-OCRL$_{mut}$. OCRL$_{mut}$ was attached with mCherry for recognition (not shown).

(**D**) F_{PH} at the plasma membrane (PM F_{PH}) or cytosol (Cytosol F_{PH}) before (Ctrl) and after blue light exposure to cells transfected with CIBN/CRY2-OCRL$_{mut}$ (mean + s.e.m, 10 cells). a.u.: artificial unit.

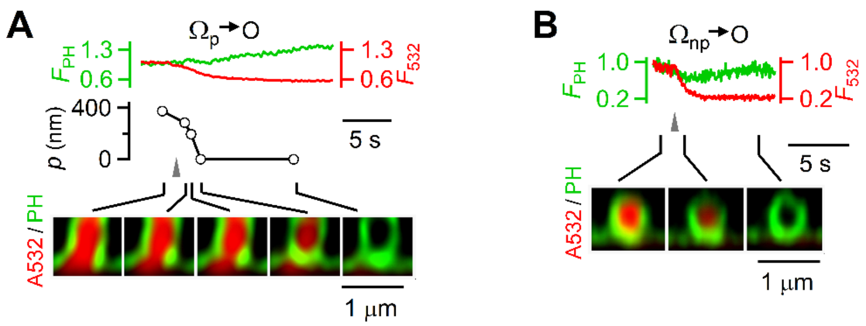

Fig. S7. STED XZ-plane imaging of pre-close events.

(**A-B**) PH$_G$ and A532 fluorescence (F_{PH}, F_{532}, normalized to baseline), and STED XZ/Y$_{fix}$ images (at times indicated with lines) for a pre-Ω undergoing pore closure.

(**A**) A preformed-Ω (pre-Ω) with a visible pore (Ω_p) that constricted and closed after depol$_{1s}$ (gray triangle). Note that this pre-Ω's pore is nearly as large as the width of the Ω-profile's body so that the Ω-profile may apparently look like an inverse U-shape.

(**B**) A pre-Ω with a non-visible pore (Ω_{np}) that closed after depol$_{1s}$ (gray triangle); pore closure was detected as F_{532} bleaching while F_{PH} sustained or decayed with a delay.

This figure is reprinted from (Ref. 10), Neuron, Volume 109, Issue 19, W. Shin, L. Wei, G. Arpino, L. Ge, X. Guo, C. Y. Chan, E. Hamid, O. Shupliakov, C. K. E. Bleck, L. G. Wu, Preformed Omega-profile closure and kiss-and-run mediate endocytosis and diverse endocytic modes in neuroendocrine chromaffin cells, 3119-3134.e5, Copyright (2021), with permission from Elsevier.

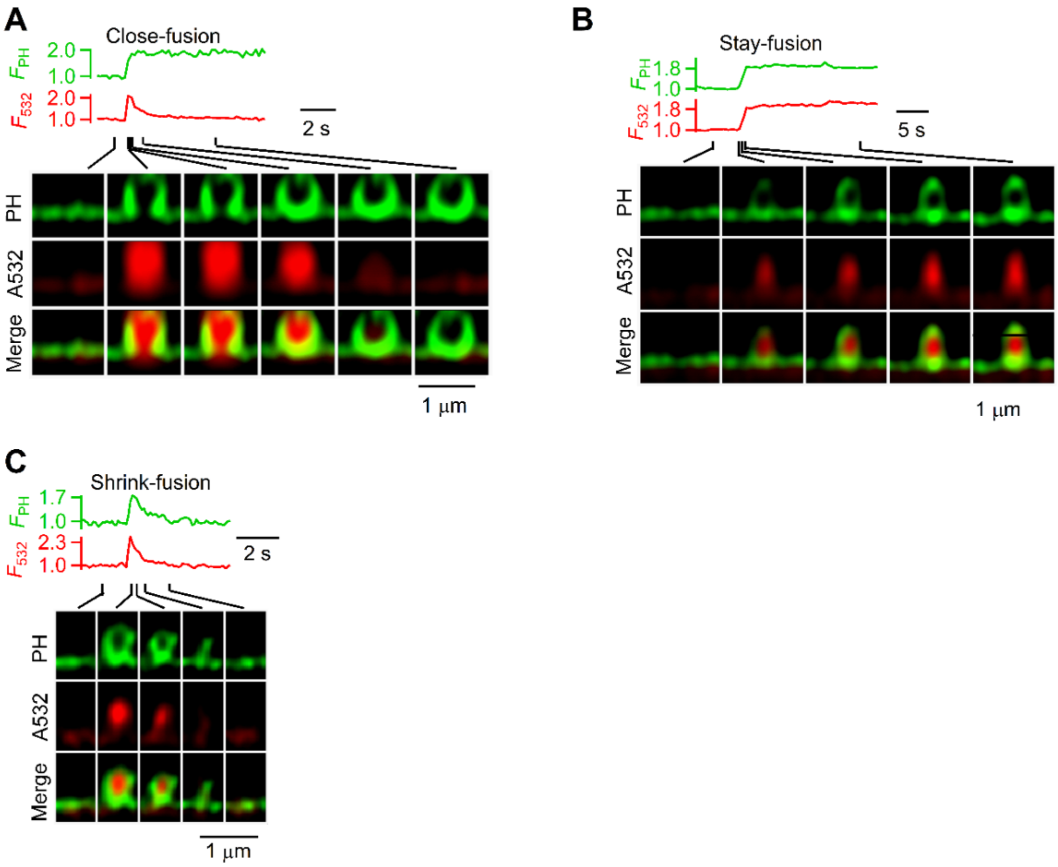

Fig. S8. Fusion-generated Ω-profiles may close its pore, maintain an open pore or shrink to merge with the plasma membrane.

(**A-C**) PH_G fluorescence (F_{PH}, normalized to baseline), A532 spot fluorescence (F_{532}, normalized to baseline), and sampled images at times indicated with lines showing that fusion-generated Ω-profiles close its pore (A, close-fusion), maintain an open pore (B, stay-fusion) or shrink to merge with the plasma membrane (C, shrink-fusion). Fusion was detected as the sudden appearance of PH_G-labelled Ω-profiles with a A532 spot within a single XZ/Y_{fix} imaging frame (every 26-200 ms) (*15*). Panel A is reprinted from (Ref. *15*), Cell, Volume 173, Issue 4, W. Shin, L. Ge, G. Arpino, S. A. Villarreal, E. Hamid, H. Liu, W. D. Zhao, P. J. Wen, H. C. Chiang, L. G. Wu, Visualization of Membrane Pore in Live Cells Reveals a Dynamic-Pore Theory Governing Fusion and Endocytosis, 934-945.e12, Copyright (2018), with permission from Elsevier; panel B and C are reprinted from (Ref. *26*), Cell Report, Volume 30, Issue 2, W. Shin, G. Arpino, S. Thiyagarajan, R. Su, L. Ge, Z. McDargh, X. Guo, L. Wei, O. Shupliakov, A. Jin, B. O'Shaughnessy, L. G. Wu, Vesicle Shrinking and Enlargement Play Opposing Roles in the Release of Exocytotic Contents, 421-431.e7, Copyright(2020), with permission from Elsevier.

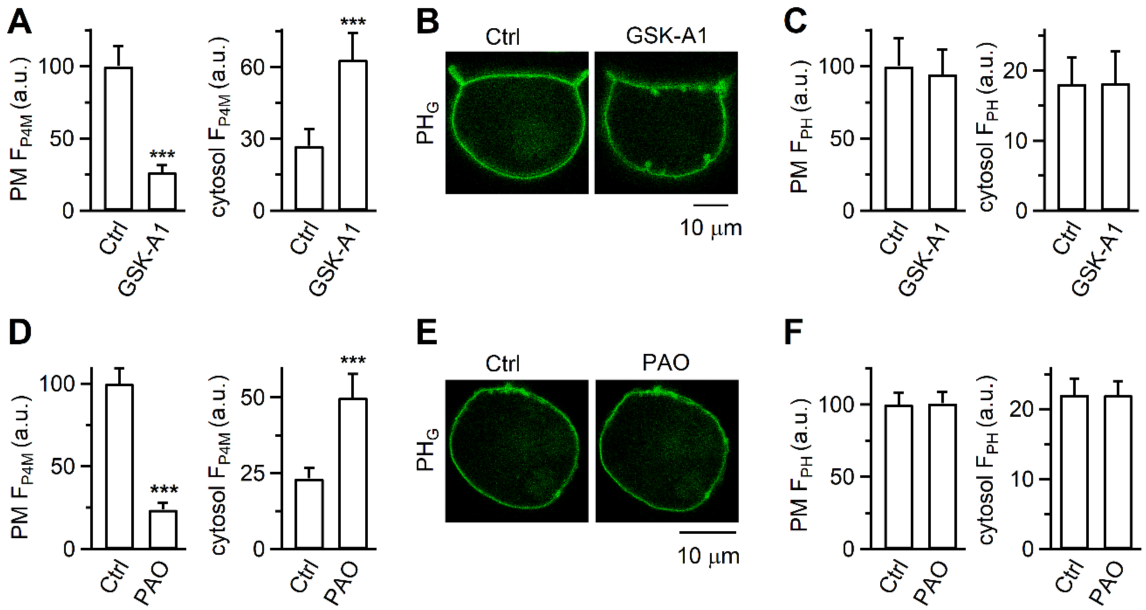

Fig. S9. GSK-A1 or PAO reduces P4M-EGFP fluorescence at the plasma membrane, increases cytosolic P4M-EGFP fluorescence, but not PH-EGFP fluorescence.

(**A**) P4M-EGFP fluorescence (F_{P4M}) at the plasma membrane (PM) and cytosol before (Ctrl) and after GSK-A1 (100 nm, 10 min) application (mean + s.e.m, 10 cells). ***: p <0.001 (t test). a.u.: arbitrary unit. Cytosol F_{P4M} increase was due to PM PI4P reduction that caused P4M-EGFP dissociation from PM to the cytosol, as reported previously in another cell type (*29*). PM F_{P4M} plot is the same as the bar graph of Figures 4A and 4B in the main text.

(**B**) Sampled PH_G images of a cell before (Ctrl) and 10 min after GSK-A1 (100 nm, 10 min) application.

(**C**) F_{PH} at PM (PM F_{PH}) or in cytosol (Cytosol F_{PH}) before (Ctrl) and 10 min after GSK-A1 (100 nm) application (mean + s.e.m, n = 10 cells). a.u.: arbitrary unit. No significant differences were observed (p > 0.05, t test).

(**D-F**) Similar arrangement as panels A-C, respectively, except PAO (20 μM, 10 min) replacing GSK-A1.

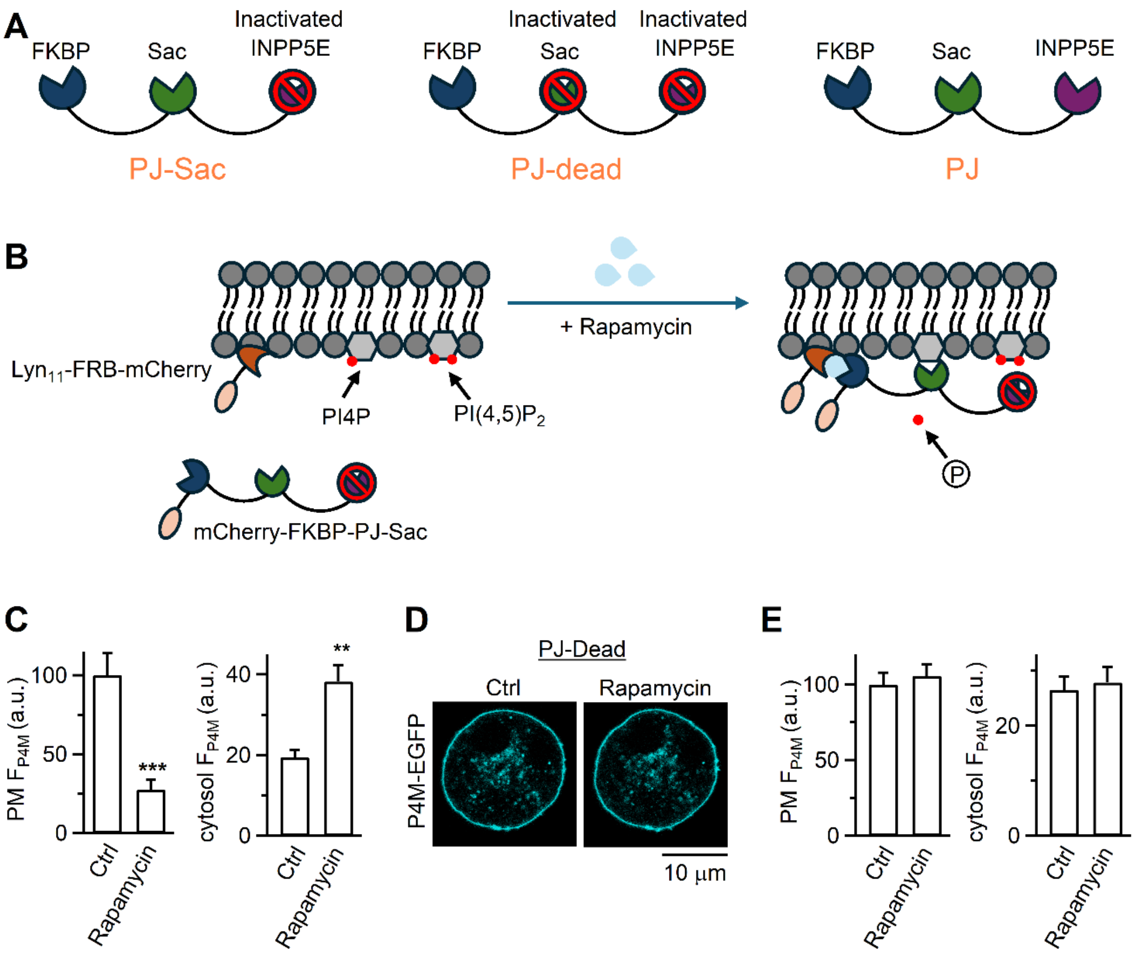

Fig. S10. Chemical-genetic approach to reduce PI4P at the plasma membrane.

(**A**) Schematic drawing showing PJ-Sac, PJ-dead and PJ construct (PJ refers to Pseudojanin).

(**B**) Schematic drawing showing PJ-Sac and the membrane targeted Lyn_{11}-FRB-mCherry construct before and after rapamycin treatment – after rapamycin treatment PI4P is dephosphorylated into PI.

(**C**) F_{P4M} at PM or in cytosol before (Ctrl) or after rapamycin application (0.5 µM, 5 min) to PJ-Sac-transfected cells (mean + s.e.m., 10 cells). **: p <0.01 (paired t test); ***: p <0.001 (paired t test). a.u.: arbitrary unit.

(**D**) Sampled P4M-EGFP images of a PJ-dead-transfected cell before (Ctrl) and after 5 min application of 0.5 µM rapamycin that causes dimerization between Lyn_{11}-FRB and FKBP-linked PJ-dead. PJ-dead: an inactivated sac1 mutant, serving as control.

(**E**) F_{P4M} at PM or in cytosol before (Ctrl) or after rapamycin application to PJ-dead-transfected cells (mean + s.e.m., 10 cells). a.u.: arbitrary unit. **: p <0.01 (paired t test); ***: p <0.001 (paired t test).

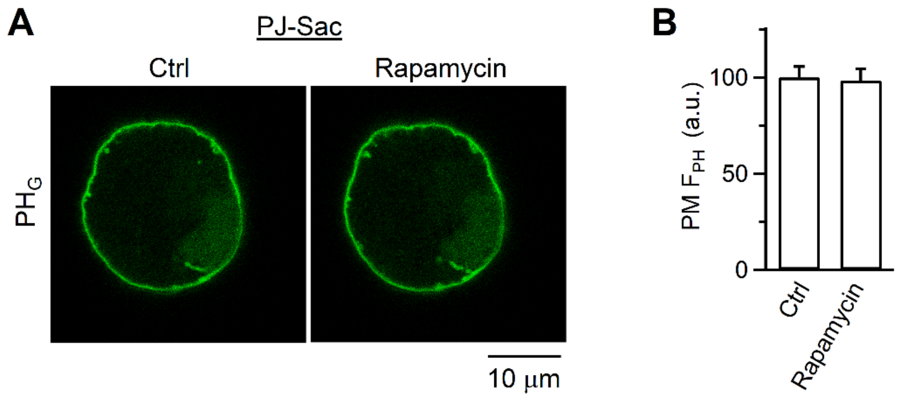

Fig. S11. Chemical-genetic approach for reducing PI4P does not affect PI(4,5)P_2 at the plasma membrane.

(**A**)Sampled PH_G (labeling PI(4,5)P_2) image of an FRB/FKBP-PJ-Sac-transfected cell before (Ctrl) and after rapamycin application (0.5 μM, 5 min).

(**B**) F_{PH} at PM before (Ctrl) and after rapamycin application (mean + s.e.m., 10 cells). a.u.: arbitrary unit. No significant differences were observed ($p > 0.05$, paired t test).

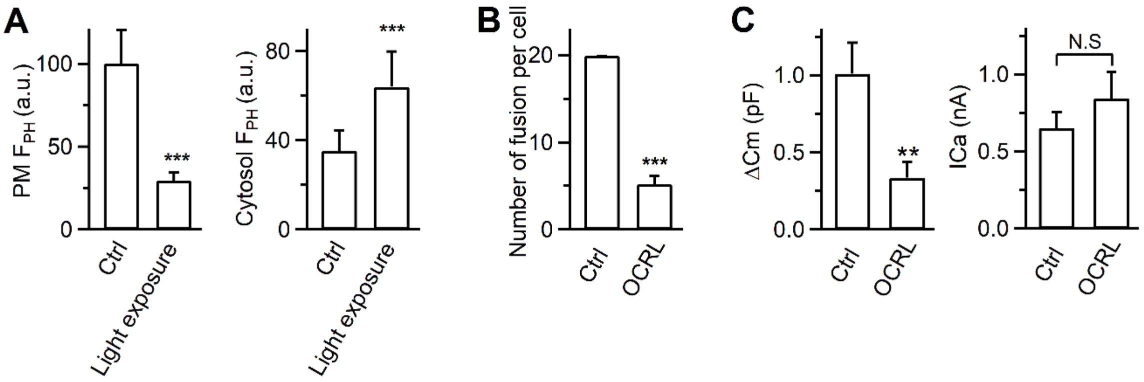

Fig. S12. Optogenetic reduction of $PI(4,5)P_2$ induces an increase of the cytosolic F_{PH} in cells transfected with CIBN-CAAX/CRY2-OCRL.

(**A**) PM (plasma membrane) and cytosol F_{PH} before (Ctrl) and after a blue light exposure in cells transfected with mCherry-attached CIBN-CAAX/CRY2-OCRL (mean + s.e.m., 10 cells). a.u.: artificial unit. ***: $p < 0.001$, paired t test. Cytosol F_{PH} increase was due to the reduction of $PI(4,5)P_2$ at the plasma membrane that caused PH_G dissociation from the plasma membrane into the cytosol. This plot is identical to Fig. S6B. We replot it here for readers' convenience.

(**B**) Number of fusion spots (per cell bottom, confocal XY-plane imaging, mean + s.e.m.) induced by $depol_{1s}$ in Ctrl (11 cells) or CIBN/CRY2-OCRL-transfected cells exposed with blue-light (11 cells).

(**C**) ΔCm and ICa (mean + s.e.m.) induced by $depol_{1s}$ in control (Ctrl, 14 cells) or in CIBN-CAAX/CRY2-OCRL-transfected cells exposed with blue light (13 cells).

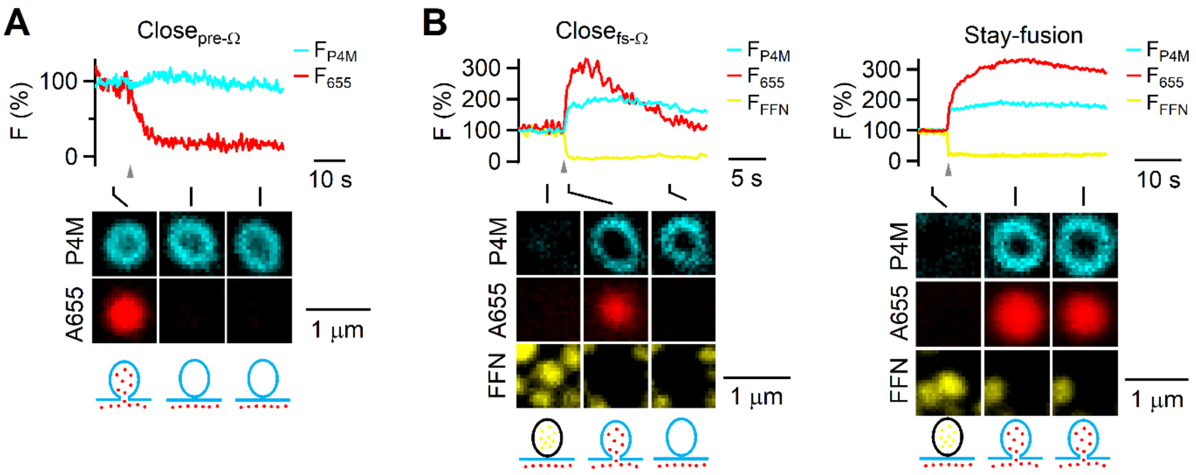

Fig. S13. Detection of Close$_{pre-\Omega}$ and Close$_{fs-\Omega}$ by replacing PH$_G$ with P4M-EGFP.

(**A**) F$_{P4M}$, F$_{655}$, and sampled confocal XY-plane images at times indicated by lines for depol$_{1s}$ (gray triangle)-induced preformed-Ω pore closure (Close$_{pre-\Omega}$). A carton drawing at the bottom illustrates the pore closure process.

(**B**) F$_{P4M}$, F$_{655}$, F$_{FFN}$, and sampled confocal XY-plane images showing close-fusion (left, Close$_{fs-\Omega}$) and stay-fusion (right). Carton drawings at the bottom illustrate the fusion pore dynamics.

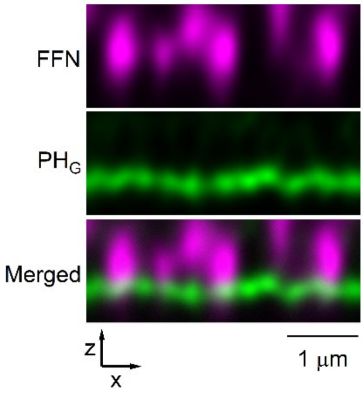

Fig. S14. PI(4,5)P_2 at the vesicle docking site of the plasma membrane.

XZ-plane STED images of FFN511 (upper) and PH_G [middle, labeling PI(4,5)P_2] in a chromaffin cell (images merged at the bottom).

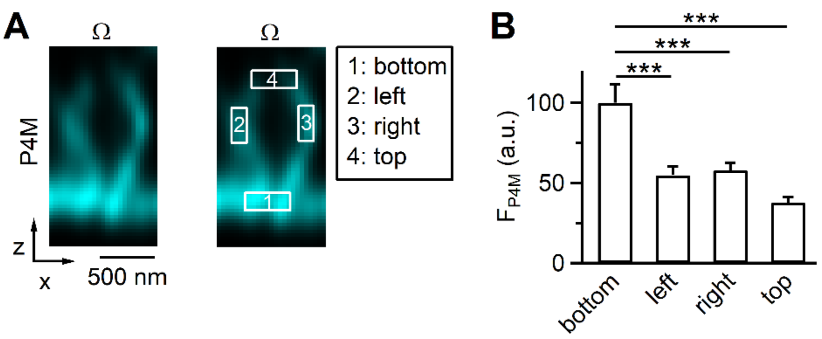

Fig. S15. P4M-EGFP-labeled PI4P was spatially enriched at the neck of the preformed Ω-profile.

(**A**) Left: STED XZ/Y_{fix} image of P4M-EGFP showing a preformed Ω-profiles.
Right: the spatial distribution of the PI4P biosensor P4M around the Ω-profile by measuring fluorescence intensity at four defined positions: the bottom (corresponding to the vesicle neck), left, right, and top of the Ω-profile.

(**B**) P4M-EGFP fluorescence intensity (F_{P4M}, mean + s.e.m.) at the bottom, left, right, and top of the Ω-profile (18 pre-Ω, 4 cells). ***: $p < 0.001$ (t test). a.u.: arbitrary unit.

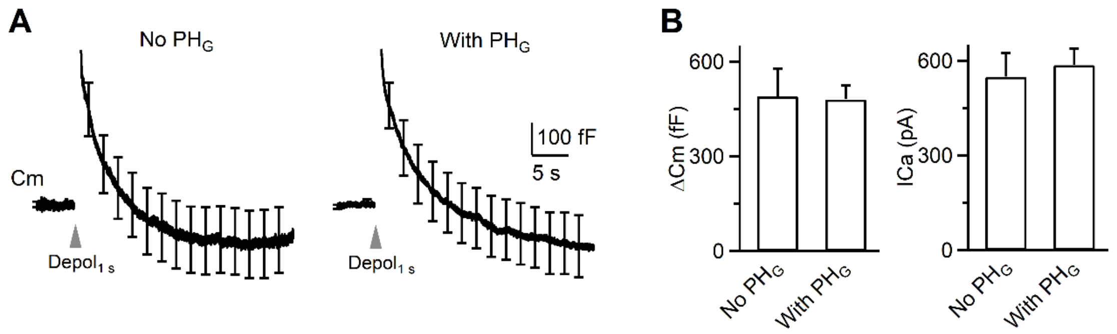

Fig. S16. Phospholipase C delta PH-domain attached with mNeonGreen (PH_G) does not affect exocytosis in chromaffin cells.

(**A**) Similar capacitance (Cm) jump (indicating exocytosis, mean ± s.e.m.) induced by $depol_{1s}$ (gray triangle) in cells with (n = 35) or without (n = 35) PH_G overexpression.

(**B**) ΔCm and peak ICa (mean + s.e.m.) induced by $depol_{1s}$ in cells with (n = 35) or without (n = 35) PH_G overexpression.

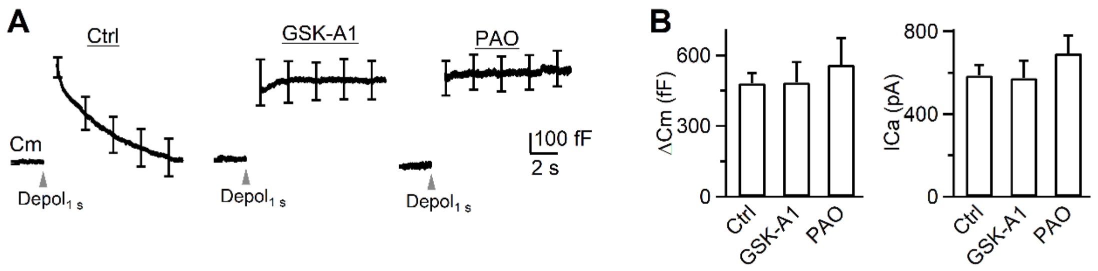

Fig. S17. GSK-A1 or PAO does not affect exocytosis.

(**A**) Averaged capacitance (Cm, mean ± s.e.m.) traces induced by $depol_{1s}$ (gray triangle) in control cells (Ctrl, n = 35), GSK-A1-treated cells (GSK-A1, n = 17), and PAO-treated cells (PAO, n = 22).

(**B**) Quantification of ΔCm and peak ICa (mean + s.e.m.) induced by $depol_{1s}$ in three groups of cells shown in panel A.

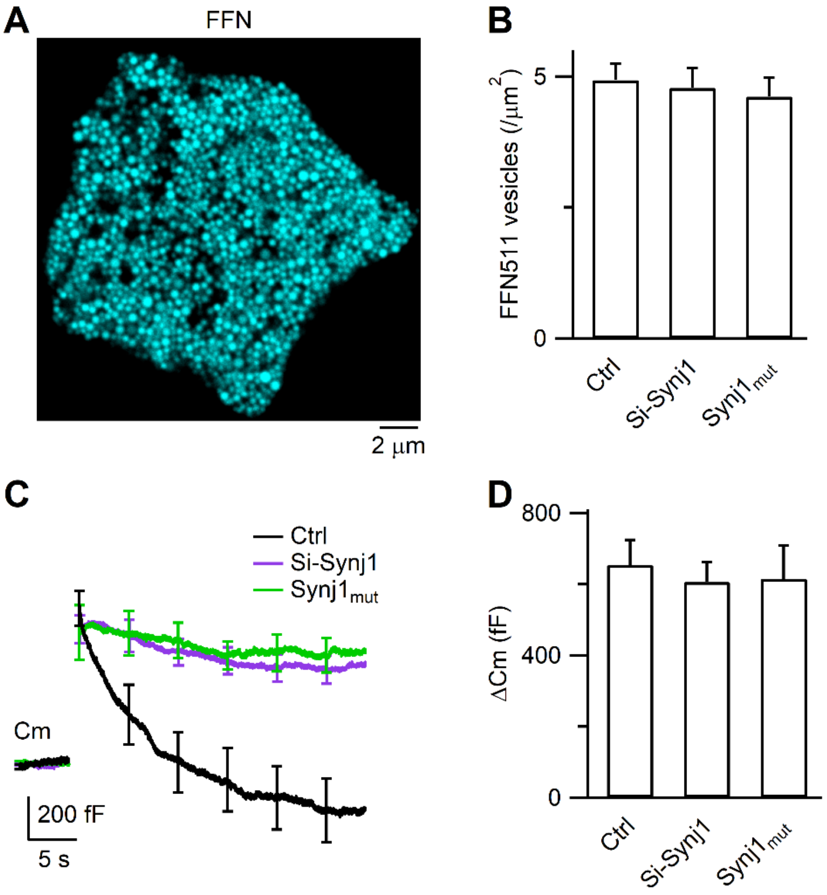

Fig. S18. Synaptojanin inhibition does not affect exocytosis.

(**A**) XY-plane confocal image at the cell bottom showing FFN511-labeled vesicles at the plasma membrane.

(**B**) The number of FFN511-labeled vesicles per square micrometer (mean + s.e.m.) under resting conditions was quantified in the confocal microscope's XY-plane for Ctrl (10 cells), Si-Synj1 (10 cells), and Synj1$_{mut}$ (10 cells). No significant differences were observed among these groups.

(**C-D**) Averaged capacitance traces (mean ± s.e.m.) and the capacitance jump (ΔCm, mean + s.e.m.) induced by depol$_{1s}$ (gray triangle) in Ctrl (25 cells), Si-Synj1 (15 cells), and Synj1$_{mut}$ (17 cells). No significant differences in ΔCm were observed among these groups (D).

REFERENCES AND NOTES

1. M. Kaksonen, A. Roux, Mechanisms of clathrin-mediated endocytosis. *Nat. Rev. Mol. Cell Biol.* **19**, 313–326 (2018).

2. N. L. Kononenko, V. Haucke, Molecular mechanisms of presynaptic membrane retrieval and synaptic vesicle reformation. *Neuron* **85**, 484–496 (2015).

3. Q. Gan, S. Watanabe, Synaptic vesicle endocytosis in different model systems. *Front. Cell. Neurosci.* **12**, 171 (2018).

4. L. G. Wu, E. Hamid, W. Shin, H. C. Chiang, Exocytosis and endocytosis: Modes, functions, and coupling mechanisms. *Annu. Rev. Physiol.* **76**, 301–331 (2014).

5. S. Watanabe, E. Boucrot, Fast and ultrafast endocytosis. *Curr. Opin. Cell Biol.* **47**, 64–71 (2017).

6. M. M. Kozlov, J. W. Taraska, Generation of nanoscopic membrane curvature for membrane trafficking. *Nat. Rev. Mol. Cell Biol.* **24**, 63–78 (2023).

7. L. G. Wu, C. Y. Chan, Membrane transformations of fusion and budding. *Nat. Commun.* **15**, 21 (2024).

8. N. L. Chanaday, M. A. Cousin, I. Milosevic, S. Watanabe, J. R. Morgan, The synaptic vesicle cycle revisited: New insights into the modes and mechanisms. *J. Neurosci.* **39**, 8209–8216 (2019).

9. H. C. Chiang, W. Shin, W. D. Zhao, E. Hamid, J. Sheng, M. Baydyuk, P. J. Wen, A. Jin, F. Momboisse, L. G. Wu, Post-fusion structural changes and their roles in exocytosis and endocytosis of dense-core vesicles. *Nat. Commun.* **5**, 3356 (2014).

10. W. Shin, L. Wei, G. Arpino, L. Ge, X. Guo, C. Y. Chan, E. Hamid, O. Shupliakov, C. K. E. Bleck, L. G. Wu, Preformed Omega-profile closure and kiss-and-run mediate endocytosis and diverse endocytic modes in neuroendocrine chromaffin cells. *Neuron* **109**, 3119–3134.e5 (2021).

11. H. Choudhry, M. Aggarwal, P. Y. Pan, Mini-review: Synaptojanin 1 and its implications in membrane trafficking. *Neurosci. Lett.* **765**, 136288 (2021).

12. K. Hardies, Y. Cai, C. Jardel, A. C. Jansen, M. Cao, P. May, T. Djemie, C. Hachon Le Camus, K. Keymolen, T. Deconinck, V. Bhambhani, C. Long, S. A. Sajan, K. L. Helbig, AR working group of the EuroEPINOMICS RES Consortium, A. Suls, R. Balling, I. Helbig, P. De Jonghe, C. Depienne, P. De Camilli, S. Weckhuysen, Loss of SYNJ1 dual phosphatase activity leads to early onset refractory seizures and progressive neurological decline. *Brain* **139**, 2420–2430 (2016).

13. O. Attree, I. M. Olivos, I. Okabe, L. C. Bailey, D. L. Nelson, R. A. Lewis, R. R. McInnes, R. L. Nussbaum, The Lowe's oculocerebrorenal syndrome gene encodes a protein highly homologous to inositol polyphosphate-5-phosphatase. *Nature* **358**, 239–242 (1992).

14. A. T. Pagnamenta, M. F. Howard, E. Wisniewski, N. Popitsch, S. J. Knight, D. A. Keays, G. Quaghebeur, H. Cox, P. Cox, T. Balla, J. C. Taylor, U. Kini, Germline recessive mutations in PI4KA are associated with perisylvian polymicrogyria, cerebellar hypoplasia and arthrogryposis. *Hum. Mol. Genet.* **24**, 3732–3741 (2015).

15. W. Shin, L. Ge, G. Arpino, S. A. Villarreal, E. Hamid, H. Liu, W. D. Zhao, P. J. Wen, H. C. Chiang, L. G. Wu, Visualization of membrane pore in live cells reveals a dynamic-pore theory governing fusion and endocytosis. *Cell* **173**, 934–945.e12 (2018).

16. W. D. Zhao, E. Hamid, W. Shin, P. J. Wen, E. S. Krystofiak, S. A. Villarreal, H. C. Chiang, B. Kachar, L. G. Wu, Hemi-fused structure mediates and controls fusion and fission in live cells. *Nature* **534**, 548–552 (2016).

17. G. R. Hammond, M. P. Machner, T. Balla, A novel probe for phosphatidylinositol 4-phosphate reveals multiple pools beyond the Golgi. *J. Cell Biol.* **205**, 113–126 (2014).

18. C. Ji, Y. Zhang, P. Xu, T. Xu, X. Lou, Nanoscale landscape of phosphoinositides revealed by specific pleckstrin homology (PH) domains using single-molecule superresolution imaging in the plasma membrane. *J. Biol. Chem.* **290**, 26978–26993 (2015).

19. F. A. Meunier, S. L. Osborne, G. R. Hammond, F. T. Cooke, P. J. Parker, J. Domin, G. Schiavo, Phosphatidylinositol 3-kinase C2alpha is essential for ATP-dependent priming of neurosecretory granule exocytosis. *Mol. Biol. Cell* **16**, 4841–4851 (2005).

20. P. J. Wen, S. L. Osborne, I. C. Morrow, R. G. Parton, J. Domin, F. A. Meunier, Ca^{2+}-regulated pool of phosphatidylinositol-3-phosphate produced by phosphatidylinositol 3-kinase C2α on neurosecretory vesicles. *Mol. Biol. Cell* **19**, 5593–5603 (2008).

21. P. Y. Pan, P. Sheehan, Q. Wang, X. Zhu, Y. Zhang, I. Choi, X. Li, J. Saenz, J. Zhu, J. Wang, F. El Gaamouch, L. Zhu, D. Cai, Z. Yue, Synj1 haploinsufficiency causes dopamine neuron vulnerability and alpha-synuclein accumulation in mice. *Hum. Mol. Genet.* **29**, 2300–2312 (2020).

22. S. Taghavi, R. Chaouni, A. Tafakhori, L. J. Azcona, S. G. Firouzabadi, M. D. Omrani, J. Jamshidi, B. Emamalizadeh, G. A. Shahidi, M. Ahmadi, S. A. H. Habibi, A. Ahmadifard, A. Fazeli, M. Motallebi, P. Petramfar, S. Askarpour, S. Askarpour, H. A. Shahmohammadibeni, N. Shahmohammadibeni, H. Eftekhari, A. E. S. Zarneh, S. Mohammadihosseinabad, M. Khorrami, S. Najmi, A. Chitsaz, P. Shokraeian, H. Ehsanbakhsh, J. Rezaeidian, R. E. Rad, F. Madadi, M. Andarva, E. Alehabib, M. Atakhorrami, S. E. Mortazavi, Z. Azimzadeh, M. Bayat, A. M. Besharati, M. A. Harati-Ghavi, S. Omidvari, Z. Dehghani-Tafti, F. Mohammadi, B. M. H. Pour, H. N. Moghaddam, E. E. Shandiz, A. Habibi, Z. Taherian-Esfahani, H. Darvish, C. Paisán-Ruiz, A clinical and molecular genetic study of 50 families with autosomal recessive Parkinsonism revealed known and novel gene mutations. *Mol. Neurobiol.* **55**, 3477–3489 (2018).

23. C. Ji, F. Fan, X. Lou, Vesicle docking is a key target of local $PI(4,5)P_2$ metabolism in the secretory pathway of INS-1 cells. *Cell Rep.* **20**, 1409–1421 (2017).

24. L. Ge, W. Shin, G. Arpino, L. Wei, C. Y. Chan, C. K. E. Bleck, W. Zhao, L. G. Wu, Sequential compound fusion and kiss-and-run mediate exo- and endocytosis in excitable cells. *Sci. Adv.* **8**, eabm6049 (2022).

25. L. Wei, X. Guo, E. Haimov, K. Obashi, S. H. Lee, W. Shin, M. Sun, C. Y. Chan, J. Sheng, Z. Zhang, A. Mohseni, S. Ghosh Dastidar, X. S. Wu, X. Wang, S. Han, G. Arpino, B. Shi, M.

Molakarimi, J. Matthias, C. A. Wurm, L. Gan, J. W. Taraska, M. M. Kozlov, L. G. Wu, Clathrin mediates membrane fission and budding by constricting membrane pores. *Cell Discov.* **10**, 62 (2024).

26. W. Shin, G. Arpino, S. Thiyagarajan, R. Su, L. Ge, Z. McDargh, X. Guo, L. Wei, O. Shupliakov, A. Jin, B. O'Shaughnessy, L. G. Wu, Vesicle shrinking and enlargement play opposing roles in the release of exocytotic contents. *Cell Rep.* **30**, 421–431.e7 (2020).

27. A. L. Leivers, M. Tallant, J. B. Shotwell, S. Dickerson, M. R. Leivers, O. B. McDonald, J. Gobel, K. L. Creech, S. L. Strum, A. Mathis, S. Rogers, C. B. Moore, J. Botyanszki, Discovery of selective small molecule type III phosphatidylinositol 4-kinase alpha (PI4KIIIα) inhibitors as anti hepatitis C (HCV) agents. *J. Med. Chem.* **57**, 2091–2106 (2014).

28. N. Bojjireddy, J. Botyanszki, G. Hammond, D. Creech, R. Peterson, D. C. Kemp, M. Snead, R. Brown, A. Morrison, S. Wilson, S. Harrison, C. Moore, T. Balla, Pharmacological and genetic targeting of the PI4KA enzyme reveals its important role in maintaining plasma membrane phosphatidylinositol 4-phosphate and phosphatidylinositol 4,5-bisphosphate levels. *J. Biol. Chem.* **289**, 6120–6132 (2014).

29. G. R. V. Hammond, M. J. Fischer, K. E. Anderson, J. Holdich, A. Koteci, T. Balla, R. F. Irvine, PI4P and PI(4,5)P-2 are essential but independent lipid determinants of membrane identity. *Science* **337**, 727–730 (2012).

30. T. F. Martin, Role of PI(4,5)P_2 in vesicle exocytosis and membrane fusion. *Subcell. Biochem.* **59**, 111–130 (2012).

31. K. C. Gwosch, J. K. Pape, F. Balzarotti, P. Hoess, J. Ellenberg, J. Ries, S. W. Hell, MINFLUX nanoscopy delivers 3D multicolor nanometer resolution in cells. *Nat. Methods* **17**, 217–224 (2020).

32. C. R. Artalejo, J. R. Henley, M. A. McNiven, H. C. Palfrey, Rapic endocytosis coupled to exocytosis in adrenal chromaffin cells involves Ca^{2+}, GTP, and dynamin but not clathrin. *Proc. Natl. Acad. Sci. U.S.A.* **92**, 8328–8332 (1995).

33. X. S. Wu, B. D. McNeil, J. Xu, J. Fan, L. Xue, E. Melicoff, R. Adachi, L. Bai, L. G. Wu, Ca^{2+} and calmodulin initiate all forms of endocytosis during depolarization at a nerve terminal. *Nat. Neurosci.* **12**, 1003–1010 (2009).

34. N. Hosoi, M. Holt, T. Sakaba, Calcium dependence of exo- and endocytotic coupling at a glutamatergic synapse. *Neuron* **63**, 216–229 (2009).

35. E. L. Clayton, G. J. Evans, M. A. Cousin, Bulk synaptic vesicle endocytosis is rapidly triggered during strong stimulation. *J. Neurosci.* **28**, 6627–6632 (2008).

36. T. Sun, X. S. Wu, J. Xu, B. D. McNeil, Z. P. Pang, W. Yang, L. Bai, S. Qadri, J. D. Molkentin, D. T. Yue, L. G. Wu, The role of calcium/calmodulin-activated calcineurin in rapid and slow endocytosis at central synapses. *J. Neurosci.* **30**, 11838–11847 (2010).

37. T. Yamashita, T. Hige, T. Takahashi, Vesicle endocytosis requires dynamin-dependent GTP hydrolysis at a fast CNS synapse. *Science* **307**, 124–127 (2005).

38. J. Xu, B. McNeil, W. Wu, D. Nees, L. Bai, L. G. Wu, GTP-independent rapid and slow endocytosis at a central synapse. *Nat. Neurosci.* **11**, 45–53 (2008).

39. S. Watanabe, B. R. Rost, M. Camacho-Perez, M. W. Davis, B. Sohl-Kielczynski, C. Rosenmund, E. M. Jorgensen, Ultrafast endocytosis at mouse hippocampal synapses. *Nature* **504**, 242–247 (2013).

40. A. Raimondi, S. M. Ferguson, X. Lou, M. Armbruster, S. Paradise, S. Giovedi, M. Messa, N. Kono, J. Takasaki, V. Cappello, E. O'toole, T. A. Ryan, P. De Camilli, Overlapping role of dynamin isoforms in synaptic vesicle endocytosis. *Neuron* **70**, 1100–1114 (2011).

41. X. Lou, S. Paradise, S. M. Ferguson, P. De Camilli, Selective saturation of slow endocytosis at a giant glutamatergic central synapse lacking dynamin 1. *Proc. Natl. Acad. Sci. U.S.A.* **105**, 17555–17560 (2008).

42. L. Xue, B. D. McNeil, X. S. Wu, F. Luo, L. He, L. G. Wu, A membrane pool retrieved via endocytosis overshoot at nerve terminals: A study of its retrieval mechanism and role. *J. Neurosci.* **32**, 3398–3404 (2012).

43. J. Leitz, E. T. Kavalali, Ca^{2+} influx slows single synaptic vesicle endocytosis. *J. Neurosci.* **31**, 16318–16326 (2011).

44. M. Wienisch, J. Klingauf, Vesicular proteins exocytosed and subsequently retrieved by compensatory endocytosis are nonidentical. *Nat. Neurosci.* **9**, 1019–1027 (2006).

45. T. Fernandez-Alfonso, R. Kwan, T. A. Ryan, Synaptic vesicles interchange their membrane proteins with a large surface reservoir during recycling. *Neuron* **51**, 179–186 (2006).

46. Y. Hua, R. Sinha, C. S. Thiel, R. Schmidt, J. Huve, H. Martens, S. W. Hell, A. Egner, J. Klingauf, A readily retrievable pool of synaptic vesicles. *Nat. Neurosci.* **14**, 833–839 (2011).

47. G. Narkis, R. Ofir, D. Landau, E. Manor, M. Volokita, R. Hershkowitz, K. Elbedour, O. S. Birk, Lethal contractural syndrome type 3 (LCCS3) is caused by a mutation in PIP5K1C, which encodes PIPKI gamma of the phophatidylinsitol pathway. *Am. J. Hum. Genet.* **81**, 530–539 (2007).

48. K. Salim, M. J. Bottomley, E. Querfurth, M. J. Zvelebil, I. Gout, R. Scaife, R. L. Margolis, R. Gigg, C. I. Smith, P. C. Driscoll, M. D. Waterfield, G. Panayotou, Distinct specificity in the recognition of phosphoinositides by the pleckstrin homology domains of dynamin and Bruton's tyrosine kinase. *EMBO J.* **15**, 6241–6250 (1996).

49. J. Zheng, S. M. Cahill, M. A. Lemmon, D. Fushman, J. Schlessinger, D. Cowburn, Identification of the binding site for acidic phospholipids on the pH domain of dynamin: Implications for stimulation of GTPase activity. *J. Mol. Biol.* **255**, 14–21 (1996).

50. B. Barylko, D. Binns, K. M. Lin, M. A. Atkinson, D. M. Jameson, H. L. Yin, J. P. Albanesi, Synergistic activation of dynamin GTPase by Grb2 and phosphoinositides. *J. Biol. Chem.* **273**, 3791–3797 (1998).

51. B. Antonny, C. Burd, P. De Camilli, E. Chen, O. Daumke, K. Faelber, M. Ford, V. A. Frolov, A. Frost, J. E. Hinshaw, T. Kirchhausen, M. M. Kozlov, M. Lenz, H. H. Low, H. McMahon, C. Merrifield, T. D. Pollard, P. J. Robinson, A. Roux, S. Schmid, Membrane fission by dynamin: What we know and what we need to know. *EMBO J.* **35**, 2270–2284 (2016).

52. M. Omar-Hmeadi, A. Gucek, S. Barg, Local PI(4,5)P_2 signaling inhibits fusion pore expansion during exocytosis. *Cell Rep.* **42**, 112036 (2023).

53. G. Di Paolo, H. S. Moskowitz, K. Gipson, M. R. Wenk, S. Voronov, M. Obayashi, R. Flavell, R. M. Fitzsimonds, T. A. Ryan, P. De Camilli, Impaired PtdIns(4,5)P_2 synthesis in nerve terminals produces defects in synaptic vesicle trafficking. *Nature* **431**, 415–422 (2004).

54. S. Bolz, N. Kaempf, D. Puchkov, M. Krauss, G. Russo, T. Soykan, C. Schmied, M. Lehmann, R. Muller, C. Schultz, D. Perrais, T. Maritzen, V. Haucke, Synaptotagmin 1-triggered lipid signaling facilitates coupling of exo- and endocytosis. *Neuron* **111**, 3765–3774.e7 (2023).

55. M. Mani, S. Y. Lee, L. Lucast, O. Cremona, G. Di Paolo, P. De Camilli, T. A. Ryan, The dual phosphatase activity of synaptojanin1 is required for both efficient synaptic vesicle endocytosis and reavailability at nerve terminals. *Neuron* **56**, 1004–1018 (2007).

56. N. Rusk, P. U. Le, S. Mariggio, G. Guay, C. Lurisci, I. R. Nabi, D. Corda, M. Symons, Synaptojanin 2 functions at an early step of clathrin-mediated endocytosis. *Curr. Biol.* **13**, 659–663 (2003).

57. O. Cremona, G. Di Paolo, M. R. Wenk, A. Luthi, W. T. Kim, K. Takei, L. Daniell, Y. Nemoto, S. B. Shears, R. A. Flavell, D. A. McCormick, P. De Camilli, Essential role of phosphoinositide metabolism in synaptic vesicle recycling. *Cell* **99**, 179–188 (1999).

58. Y. Sun, S. Carroll, M. Kaksonen, J. Y. Toshima, D. G. Drubin, PtdIns(4,5)P_2 turnover is required for multiple stages during clathrin- and actin-dependent endocytic internalization. *J. Cell Biol.* **177**, 355–367 (2007).

59. B. Chang-Ileto, S. G. Frere, R. B. Chan, S. V. Voronov, A. Roux, G. Di Paolo, Synaptojanin 1-mediated PI(4,5)P_2 hydrolysis is modulated by membrane curvature and facilitates membrane fission. *Dev. Cell* **20**, 206–218 (2011).

60. R. W. Holz, M. D. Hlubek, S. D. Sorensen, S. K. Fisher, T. Balla, S. Ozaki, G. D. Prestwich, E. L. Stuenkel, M. A. Bittner, A pleckstrin homology domain specific for phosphatidylinositol 4, 5-bisphosphate (PtdIns-4,5-P_2) and fused to green fluorescent protein identifies plasma membrane PtdIns-4,5-P_2 as being important in exocytosis. *J. Biol. Chem.* **275**, 17878–17885 (2000).

61. I. Milosevic, J. B. Sorensen, T. Lang, M. Krauss, G. Nagy, V. Haucke, R. Jahn, E. Neher, Plasmalemmal phosphatidylinositol-4,5-bisphosphate level regulates the releasable vesicle pool size in chromaffin cells. *J. Neurosci.* **25**, 2557–2565 (2005).

62. T. F. Martin, PI(4,5)P_2-binding effector proteins for vesicle exocytosis. *Biochim. Biophys. Acta* **1851**, 785–793 (2015).

63. M. Omar-Hmeadi, N. R. Gandasi, S. Barg, PtdIns(4,5)P_2 is not required for secretory granule docking. *Traffic* **19**, 436–445 (2018).

64. M. Lindau, G. Alvarez de Toledo, The fusion pore. *Biochim. Biophys. Acta* **164**, 167–173 (2003).

65. N. C. Shaner, G. G. Lambert, A. Chammas, Y. Ni, P. J. Cranfill, M. A. Baird, B. R. Sell, J. R. Allen, R. N. Day, M. Israelsson, M. W. Davidson, J. Wang, A bright monomeric green fluorescent protein derived from Branchiostoma lanceolatum. *Nat. Methods* **10**, 407–409 (2013).

66. A. Tomas, B. Yermen, R. Regazzi, J. E. Pessin, P. A. Halban, Regulation of insulin secretion by phosphatidylinositol-4,5-bisphosphate. *Traffic* **11**, 123–137 (2010).

67. M. Lindau, E. Neher, Patch-clamp techniques for time-resolved capacitance measurements in single cells. *Pflugers Arch.* **411**, 137–146 (1988).

68. K. L. Engisch, M. C. Nowycky, Compensatory and excess retrieval: Two types of endocytosis following single step depolarizations in bovine adrenal chromaffin cells. *J. Physiol.* **506**, 591–608 (1998).

69. D. Perrais, I. C. Kleppe, J. W. Taraska, W. Almers, Recapture after exocytosis causes differential retention of protein in granules of bovine chromaffin cells. *J. Physiol.* **560**, 413–428 (2004).

70. C. Smith, E. Neher, Multiple forms of endocytosis in bovine adrenal chromaffin cells. *J. Cell Biol.* **139**, 885–894 (1997).

71. R. Schmidt, T. Weihs, C. A. Wurm, I. Jansen, J. Rehman, S. J. Sahl, S. W. Hell, MINFLUX nanometer-scale 3D imaging and microsecond-range tracking on a common fluorescence microscope. *Nat. Commun.* **12**, 1478 (2021).

72. E. M. Mulhall, A. Gharpure, R. M. Lee, A. E. Dubin, J. S. Aaron, K. L. Marshall, K. R. Spencer, M. A. Reiche, S. C. Henderson, T. L. Chew, A. Patapoutian, Direct observation of the conformational states of PIEZO1. *Nature* **620**, 1117–1125 (2023).

73. J. K. Pape, T. Stephan, F. Balzarotti, R. Buchner, F. Lange, D. Riedel, S. Jakobs, S. W. Hell, Multicolor 3D MINFLUX nanoscopy of mitochondrial MICOS proteins. *Proc. Natl. Acad. Sci. U.S.A.* **117**, 20607–20614 (2020).

74. L. Kong, K. A. Sochacki, H. Wang, S. Fang, B. Canagarajah, A. D. Kehr, W. J. Rice, M. P. Strub, J. W. Taraska, J. E. Hinshaw, Cryo-EM of the dynamin polymer assembled on lipid membrane. *Nature* **560**, 258–262 (2018).

75. J. Schindelin, I. Arganda-Carreras, E. Frise, V. Kaynig, M. Longair, T. Pietzsch, S. Preibisch, C. Rueden, S. Saalfeld, B. Schmid, J. Y. Tinevez, D. J. White, V. Hartenstein, K. Eliceiri, P. Tomancak, A. Cardona, Fiji: An open-source platform for biological-image analysis. *Nat. Methods* **9**, 676–682 (2012).

76. K. Soda, D. M. Balkin, S. M. Ferguson, S. Paradise, I. Milosevic, S. Giovedi, L. Volpicelli-Daley, X. Tian, Y. Wu, H. Ma, S. H. Son, R. Zheng, G. Moeckel, O. Cremona, L. B. Holzman,

P. De Camilli, S. Ishibe, Role of dynamin, synaptojanin, and endophilin in podocyte foot processes. *J. Clin. Invest.* **122**, 4401–4411 (2012).
